# Research Progress on Personalized Bone Implants Based on Additive Manufacturing

**DOI:** 10.3390/mi16121339

**Published:** 2025-11-27

**Authors:** Bingwei Gao, Zhonghui Sun, Yanquan Tong, Hongtao Yu, Feng Wang

**Affiliations:** 1 School of Mechanical and Power Engineering, Harbin University of Science and Technology, Harbin 150080, China; gaobingwei@hrbust.edu.cn (B.G.); sunzhonghuiszd@163.com (Z.S.); tongyanquan223@163.com (Y.T.); 2 Department of Radiology, Harbin·242 Hospital, Harbin 150066, China; wangfeng19941997@163.com

**Keywords:** bone implants, CT imaging, additive manufacturing, material properties, artificial intelligence

## Abstract

Personalized bone implants based on additive manufacturing are gradually emerging as a significant development direction in orthopedic restoration. By precisely matching anatomical structures and the functional requirements of patients, they demonstrate notable clinical advantages. Research progress in structural design, material selection, fabrication processes, and performance evaluation has been systematically outlined around the core research chain of personalized bone implants. Personalized design methods, material selection criteria, and the applicability of different additive manufacturing processes are analyzed in detail. The performance indicators of the implant are further comprehensively evaluated. The promoting role of multi-performance materials and intelligent manufacturing technology in complex bone repair functions has been revealed. The development of artificial intelligence transforms clinical data into long-term performance prediction models, further driving in-depth research in this field.

## 1. Introduction

The skeleton forms the rigid framework of the human body. It not only provides support for the body but also serve as the foundation for movements such as standing, running, and jumping. In addition to external injuries caused by sports, accidents, and other factors, internal injuries such as tumors and congenital deformities can also easily lead to bone defects, posing a threat to human health [1]. In clinical practice, artificial bone grafting is commonly used to treat bone defects because of limited autologous bone resources [2]. However, traditional standardized bone implants suffer from poor anatomical matching, suboptimal mechanical adaptation, and low osseointegration efficiency. Consequently, clinical applications demand personalized and precise bone repair techniques.

Through the collaborative advancement of computed tomography (CT) technology and Materialise’s interactive medical image control system (Mimics), personalized prosthetic bone design can be achieved based on patient imaging data. The external contour of the engineered bone closely resembles that of the defective bone, with perfect anatomical matching and restored biomechanical function. Thus, the success rate of defective bone repair is enhanced. Gao et al. [3] extracted CT data from the hip region of adult patients, and the femur and acetabulum were segmented using threshold segmentation and region growing. The femur underwent computational processing and smoothing to ensure the prosthesis possesses individualized characteristics and higher rationality. By integrating patients’ X-ray image data, Zhang et al. [4] developed a rapid reconstruction technique based on statistical shape models, and the femoral geometric features of individual patients were rapidly digitally reconstructed, which significantly improved the efficiency of three-dimensional reconstruction of defective bone.

The interior of natural bone exhibits a porous and gradient structure. To avoid stress shielding, artificial bone must possess an external contour and porous structure similar to that of autologous bone. However, traditional model fabrication techniques are difficult to implement. With the advent of additive manufacturing technology, the limitations of traditional manufacturing have been overcome. Complex geometries can be formed, implants can be customized, and the fabrication process for gradient structures has been enhanced. Zhang et al. [5] demonstrated that NiTi alloy porous scaffolds are jointly determined by the process parameters of Laser Powder Bed Fusion (L-PBF) and the structural dimensions of the bone scaffold. Furthermore, the microstructure, phase transformation temperature, and mechanical properties of the implant could be regulated through an optimized process targeting its characteristic dimensions. Onal et al. [6] designed two gradient structures, one with pore sizes decreasing from the interior to the exterior and the other with pore sizes increasing from the interior to the exterior. These structures were fabricated using Selective Laser Melting (SLM) technology, and mechanical and biological performance tests were conducted on both structures, which showed that the former exhibited superior performance metrics.

Key technological aspects of personalized bone implants are prioritized, such as design methodologies, material systems, fabrication processes, and performance evaluation. Design approaches for diverse porous structures in bone implants are systematically reviewed, the different application fields of various materials are analyzed, the auxiliary manufacturing capabilities of additive manufacturing technologies are explored, and their performance metrics are further compared and analyzed. The constraints of traditional technology have been broken by artificial intelligence, gradually evolving from static implants into dynamic data generation systems. It provides a feasible solution for ideal personalized bone grafting, achieving mathematical model prediction and postoperative real-time monitoring.

## 2. Design Method for Personalized Bone Implants

The design of personalized bone implants is divided into two parts. On the one hand, personalized design based on medical imaging achieves morphological adaptation between the implant and the autologous bone interface through CT data acquisition, segmentation, and three-dimensional reconstruction. On the other hand, the design of the internal porous structure and its gradient distribution enables the implant’s elastic modulus to closely match that of autologous bone, thereby preventing stress shielding and achieving biological functionality.

### 2.1. Medical Imaging and 3D Modeling

Medical imaging and 3D modeling serve as the core bridge for realizing the transition of personalized bone implants from concept to clinical application. By reconstructing the patient’s skeletal anatomy and defect structure, a digitally customized foundation for the implant is established, so that the limitations of poor fit associated with traditional standardized implants are overcome. It can empower biomimetic designs with highly matched morphologies, mechanically optimized porous structures, and functional interfaces that promote osseointegration. Through preoperative simulation and customized surgical guides, the precision and safety of surgical procedures have been enhanced, ultimately revolutionizing bone repair from generic approaches to precision and functional outcomes. Zhao et al. [7] employed the medical image processing tool Mimics 20.0 in conjunction with the reverse engineering software Geomagic Studio 2013 to accomplish three-dimensional skeletal reconstruction. Three-dimensional digital modeling of the fractured femoral structure was successfully achieved using Mimics software, which provides a reference for integrating clinical medical experiments with computer reverse engineering software. To overcome the inefficiency and inflexibility of traditional femoral modeling, Jin et al. [8] developed a reverse parametric method using spatial transformation and Creo. This approach, driven by key anatomical parameters, was validated to reduce prosthesis design time from weeks to days while achieving model errors below 0.3%, thus meeting the requirements for subsequent analysis and design. Li et al. [9] collected CT image data before and after surgery from a patient and constructed a three-dimensional model of the knee joint using image segmentation technology. Three-dimensional models of the meniscus and ligaments were constructed using the contour extension method, and the feasibility of this modeling approach was validated through finite element numerical simulations. Wu et al. [10] reconstructed a three-dimensional spinal model using Mimics software and designed directional guide tubes with fixed orientations on its surface, thereby the entity of the spinal guide tube was obtained through 3D printing technology. Feng et al. [11] proposed a stepwise design method based on Mimics and 3-Matic software and reconstructed spinal models to obtain 3D navigation templates. This method could be used for cortical bone trajectory fixation, with a simple and convenient design process. Matsko et al. [12] proposed a method for designing and manufacturing dental implants based on initial root scan data from patients. Through lattice structure modeling and additive manufacturing technology, root-like implant simulators featuring a fully lattice structure could be semi-automatically produced. And bone healing could be accelerated while maintaining load-bearing strength.

Personalized modeling of bone implants based on medical imaging and reverse engineering technology has established a standardized workflow encompassing CT data acquisition, three-dimensional reconstruction, finite element analysis, and surgical planning. The physical navigation template can be fabricated via 3D printing, which significantly enhances the precision and efficiency of orthopedic treatment. Current research remains focused on macro-level parameterized modeling and fundamental process optimization, with insufficient capability to reconstruct the subtle anatomical features of individual patients. To achieve accurate reduction in complex morphology and microstructure, artificial intelligence (AI)-driven algorithm technology has begun to be used to reconstruct anatomical structure and performance prediction, thereby providing a more reliable digital foundation for fully personalized implant design and surgical treatment.

### 2.2. Design Method for Porous Structures of Implants

**Figure 1:**
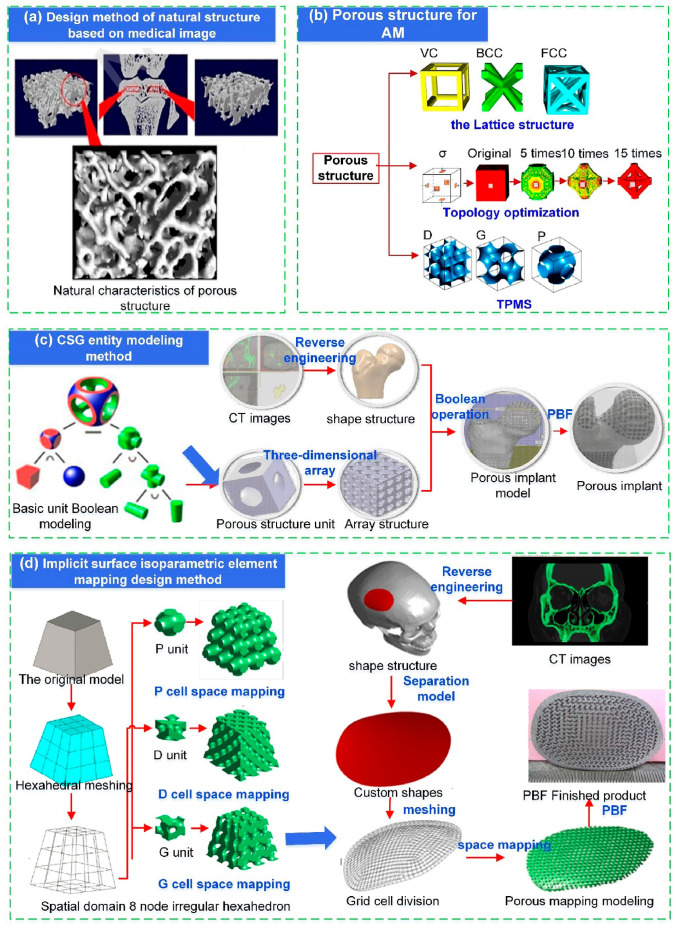
Design Methods for Porous Structures. (**a**) Direct design of porous structure based on medical image; (**b**) Typical element for the indirect design of a porous structure; (**c**) Application of the CGS solid modeling method; (**d**) Implicit surface isoparametric element mapping design method. (Reproduced from [16] with permission).

The core challenge for bone implants is mechanical compatibility, which means avoiding stress shielding, and porous structures are widely used in artificial implants. However, porous structure is not the only decisive factor. For example, there are differences in the osteogenic response between bioactive ceramics and bioinert polymers. Through complex interactions with material factors, the integration of bone and implant can be enhanced, thereby the risk of bone resorption and implant loosening is minimized [13,14,15]. Additionally, the effective surface chemistry and mechanical integrity of the porous structure are influenced by the manufacturing process, which may affect the stress between the implant and the surrounding tissue. Direct design based on medical images and indirect design based on parametric porous units are the mainstream approaches in current porous structure design. These can be subdivided into Constructive Solid Geometry (CSG) methods, topological porous structure methods, and Triple Periodic Minimal Surface (TPMS) methods, as shown in Figure 1 [16]. Three methods are systematic and widely used to create functional porous implants. They provide a comprehensive overview of the field’s main design philosophies: CSG for its intuitive control over biomimetic gradients, topology optimization for its performance-driven material layout, and TPMS for its mathematically defined, highly permeable architectures.

#### 2.2.1. Constructive Solid Geometry Method

The skeletal structure of natural bones is essentially a functional gradient material, with a continuous variation in structure and density from the dense cortical bone to the porous cancellous bone. The CSG method, while rooted in standard CAD practice, is leveraged in bone implant design for its unique capability to construct biomimetic and functionally graded porosity. By integrating 3D computer graphics and CAD modeling, body elements undergo Boolean operations to construct intricate porous structure models. Its primary application in this field lies in mimicking the transitional structure from dense cortical bone to porous cancellous bone, thereby approximating the mechanical environment of the anatomical region.

**Figure 2:**
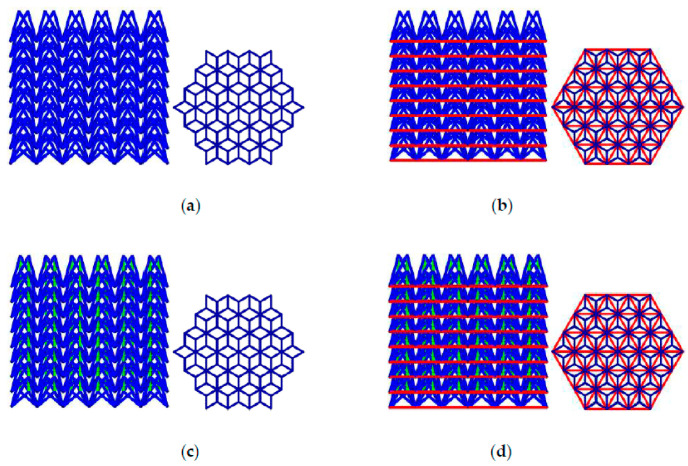
Structures fabricated by four novel NPR cells. (**a**) Type A NPR structure; (**b**) Type B NPR structure; (**c**) Type C NPR structure; (**d**) Type D NPR structure. (Reproduced from [18] with permission).

Researchers can precisely manipulate the fundamental lattice units and geometric parameters that constitute the porous structure using the CSG method, thereby controlling the effective elastic modulus and strength of the entire implant are precisely controlled. Peng et al. [17] proposed a “layered design” strategy to more accurately simulate the structural characteristics of cortical and cancellous bone. The traditional three-dimensional unit cell construction method was replaced by a two-dimensional design approach. The layered rod-connected hexagonal porous structure (LSRCMS) was constructed, and experiments demonstrated that the mechanical properties of LSRCMS closely resemble those of bone tissue. Chen [18] developed three novel Negative Poisson’s Ratio (NPR) structures based on the traditional NPR structure (Type A) by incorporating ribs in different positions, namely Types B, C, and D, which improved stiffness and energy absorption capacity. The above four structures are shown in Figure 2, where blue represents the original NPR model, green indicates the addition of ribs on the upper layer, and purple indicates the addition of ribs on the lower layer. Limmahakhun et al. [19] designed a cylindrical octahedral porous structure using CSG methods to construct a cellular CoCr implant, and experimental results demonstrated that this implant exhibits excellent mechanical properties. Shi et al. [20] proposed a design method for center and sphere gradient porous structures based on the CSG method. Gradient-porous structures and uniform-porous structures were fabricated using SLM technology, and the mechanical properties of the gradient-porous structures were demonstrated to be superior to those of the uniform-porous structures. Li et al. [21] proposed a method using spheres as hole-forming units, which performs a Boolean difference operation between spheres and cubes to generate cubic units with spherical voids. Based on a hierarchical design concept, both the inner and outer layers of the scaffold model were assigned porous elements to achieve a porous scaffold design. The research results indicated that this scaffold meets the requirements for clinical implantation. Kuhn et al. [22] designed a bone implant based on an open or partially open foam structure. The porous structure not only promoted inward bone growth but was also coated with hydroxyapatite to further stimulate osseointegration, providing a solution for personalized reconstruction in areas such as the craniofacial region.

The CSG method has revolutionized the field of bone implants through its powerful geometric modeling capabilities and seamless integration with 3D printing technology. However, the CSG method possesses inherent limitations for bone implant design. Its fidelity to the complex, stochastic microarchitecture of true trabecular bone is inherently limited. It relies on the repetition of predefined and simple geometric units. While CSG is suitable for creating ordered gradients, it is difficult to replicate biomechanically optimized irregular natural trabecular bone, and it is difficult to guarantee that the resulting structure achieves the optimal stress path. Frontier sciences such as bioactive molecules and immune regulation are expected to be integrated into future research, potentially leading to the development of intelligent bone implants that actively guide and accelerate tissue regeneration [23,24].

#### 2.2.2. Topology-Optimization-Based Design of Porous Bone Implants

**Figure 3:**
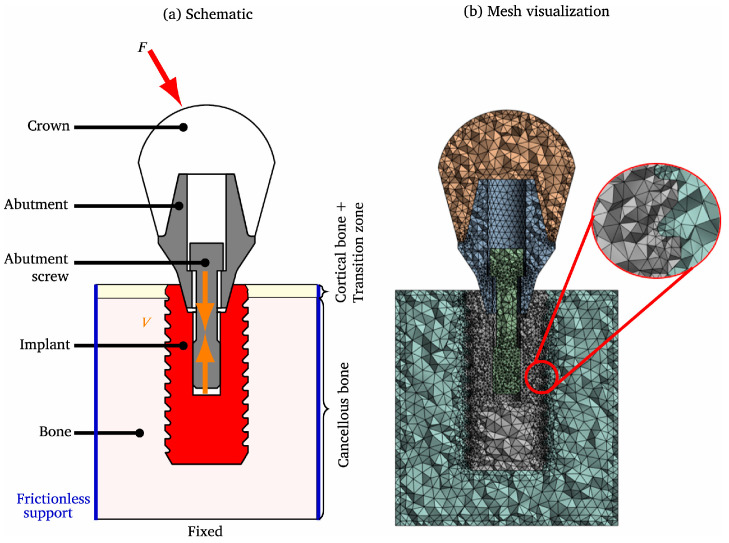
Simplified dental implant model. (**a**) Schematic representation and (**b**) mesh visualization of the ANSYS-model to simulate the stress-shielding effect. (Reproduced from [26] with permission).

The principle of topology optimization involves the optimal distribution of structural material within a continuous material domain and under known boundary conditions, thereby achieving optimal material properties such as strength, stiffness, and stress. By employing design methods based on topology optimization, structures can be engineered to achieve optimal configurations that reduce overall implant stiffness, thereby effectively minimizing stress shielding. While maintaining mechanical properties, the weight of the implant has been reduced, and the use of raw materials has been minimized. Sun et al. [25] proposed a topology optimization method based on gradient porous structures with implicit surfaces to achieve controlled shape changes in single-cell porous structures. By introducing the Heaviside function, a gradient porous structure with higher-order continuity was constructed, and this method was demonstrated to effectively regulate the properties of porous structures. Kök et al. [26] established simplified dental implant models to study how to improve their service life and performance, as shown in Figure 3. The results showed that the stress masking effect of topology-optimized implants was significantly reduced than that of uniform implants. Zhang et al. [27] proposed a novel TPMS element design method based on a T-spline-guided topology optimization approach and a dual-offset strategy, whose effectiveness was demonstrated through numerical examples. Du et al. [28] established a topology optimization model with stiffness and negative Poisson’s ratio as optimization objectives, which was solved using an improved optimality criteria (OC) algorithm. Through simulation analysis and compression tests on porous structures, it was verified that topology optimization can be applied to enhance crash-resistant load-bearing structures. Kim et al. [29] achieved synergistic optimization of mechanical properties and biological functionality through topologically optimized gradient porous structures. Its irregular porous structure on the outer surface provided initial mechanical anchoring, while the regular porous structure on the inner surface promoted osseointegration. By integrating additive manufacturing technology, lightweight high-strength components were realized, and the stress shielding effect was effectively reduced. Zhang et al. [30] proposed a personalized femoral stem design method based on topology optimization, and the optimal balance between mechanical properties and lightweighting was achieved through radial gradient porous structure design.

Topology optimization technology has now become a key driver propelling implants toward personalized, lightweight, and biofunctional designs. Intelligently distributing materials enables implants to better integrate into the human body, which is a complex mechanical and biological environment. The clinical potential of this approach is demonstrated through its application to complex anatomical sites, such as mandibular reconstruction, acetabular components, and spinal fusion cages. From structural design to clinical research on animal models, topology optimization is expected to help people create the next generation of bone implants that are smarter and more bionic.

#### 2.2.3. Design Method for Triply Periodic Minimal Surfaces

**Figure 4:**
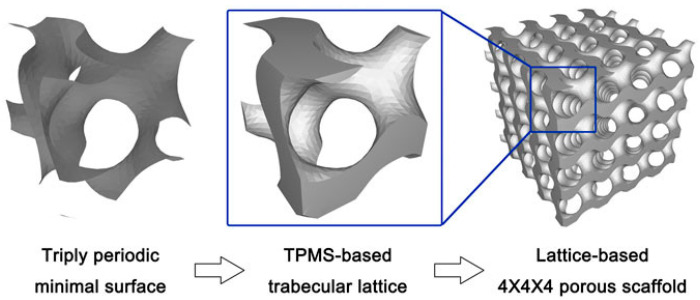
The design process of TPMS-based porous scaffolds. (Reproduced from [32] with permission).

TPMS has garnered significant attention in the field of bone implants due to its unique, highly ordered, continuously smooth, and highly interconnected porous structure. TPMS is mathematically defined surfaces with an average curvature of zero that repeat periodically in three independent directions. This geometric characteristic results in smooth, non-self-intersecting pores that are highly conducive to cell migration, nutrient transport, and bone ingrowth, making them particularly attractive for bone scaffold applications [31]. The principle of this method involves regulating the pore structure by altering parameter values within the implicit equation. Current research focuses primarily on functional gradient design and innovative structural design, and implant performance is further enhanced by integrating TPMS with topology optimization techniques. Lv et al. [32] designed multiple porous structure models using the TPMS method, as shown in Figure 4. Their mechanical properties were characterized, and the feasibility of their use as implants for different orthopedic sites was verified. Liu et al. [33] constructed three types of curved trabecular structures: S-P type, D type, and G type. Simulation results indicated that all three trabecular structures met the design requirements for human bone tissue scaffolds, which provides new insights for the surface structure design of bone implants. To investigate the mechanical performance advantages of deformed TPMS structures, Shi et al. [34] designed and fabricated porous structures of deformed G-surfaces and conventional G-surfaces with varying porosities. Compression testing confirmed that the mechanical properties of the deformed G-curve porous structure were better than those of the conventional G-curve porous structure at the same porosity. Li et al. [35] designed and fabricated a TC4 porous structure intervertebral fusion device based on TPMS and validated its performance, which was found to exhibit excellent bending resistance and biocompatibility. Fang et al. [36] designed G-type porous structures with gradient porosity and uniform G-type porous structures, and conducted comparative compression simulations on both structures. Compared to uniform structures, G-structures with radial porosity gradients had higher elastic moduli and yield strengths, and their load dispersion and transmission efficiency were effectively enhanced. Promoppatum [37] et al. designed a bone implant utilizing interconnected TPMS porous structures and mathematically controllable personalized designs. By precisely regulating its physical properties like pore size and elastic modulus, the bone implant was endowed with mechanical and biological characteristics matching those of natural bone. Chen et al. [38] achieved a three-dimensional gradient pore distribution through TPMS, and the elastic modulus of the bone tissue was precisely matched, which significantly outperformed traditional implants in energy absorption capacity and stress shielding suppression.

Through its mathematical precision, structural biomimicry, and performance tunability, the TPMS design methodology enables the fabrication of ideal bone implants. By integrating cutting-edge technologies such as gradient design, topology optimization, and multi-material printing, TPMS bone implants are anticipated to evolve toward smarter, more bionic, and more efficient solutions.

#### 2.2.4. Comparative Analysis of Design Methods

CSG methods utilize fundamental geometric units to achieve complex spatial configurations through efficient Boolean operations, excelling particularly in constructing porous structures with biomimetic gradient characteristics. Topology optimization methods are grounded in mathematical optimization principles, the optimal configuration of material distribution is achieved under specified boundary conditions, and the mechanical performance and lightweighting level of the structure are enhanced. Leveraging implicit mathematical functions, the TPMS method can generate periodic pore structures with high continuity, connectivity, and surface smoothness, which show unique advantages in terms of biocompatibility and permeability. In the study of porous structure design for bone implants, three distinct approaches address the synergy between structure and function from different perspectives. A comparative analysis is presented in Table 1. The three approaches are not mutually exclusive, and the future trend is likely to move toward their integrated use.

## 3. Material Systems for Personalized Bone Implants

To better adapt to the human body environment and achieve functional bone repair, the materials used in bone implants have undergone evolution, transitioning from simple substitutes to those that form a tighter bond with bone tissue. Biologically inert materials, such as metals and ceramics, were initially used as physical supports in many weight-bearing areas [39]. Bioactive materials represented by hydroxyapatite (HA) and calcium phosphate-based ceramics have achieved more stable chemical bonding with bone tissue [40]. To match the self-repairing capabilities of human bone tissue, biodegradable materials such as polylactic acid (PLA) and β-tricalcium phosphate (β-TCP) have emerged [41]. At present, bone implant materials are evolving toward personalization, intelligence, and functionality. Tissue-inductive biomaterials represent the cutting edge of biomaterials science, with the goal of achieving the regeneration and reconstruction of human tissues or organs. This evolution is particularly evident in metallic biomaterials. For instance, in biodegradable magnesium (Mg) implants, advanced coating strategies are being developed to control the degradation rate. Research on the wettability and reactivity of liquid magnesium with metallic substrates (e.g., silver) provides critical insights for designing novel, adherent coatings [42]. Concurrently, vivo studies are clarifying the complex tissue responses to Mg-based implants, paving the way for their clinical translation [43].

### 3.1. Metal Materials

Due to its excellent mechanical properties and load-bearing capabilities, metal has long been the preferred biomaterial for repairing damaged hard bone tissue. The first generation of bone implant materials comprised biologically inert substances such as titanium alloys, cobalt-chromium alloys, and stainless steel, which remain the most commonly used materials today. Due to its lightweight, non-magnetic, non-toxic properties, corrosion resistance, and high strength, titanium and titanium alloys are considered ideal materials for human implants and continue to be used in many weight-bearing applications to this day [44]. Cobalt-chromium alloys have become the material of choice for joint prostheses due to their exceptional wear and corrosion resistance. Tantalum exhibits outstanding osseointegration properties, earning it the designation of a “biocompatible metal”.

**Figure 5:**
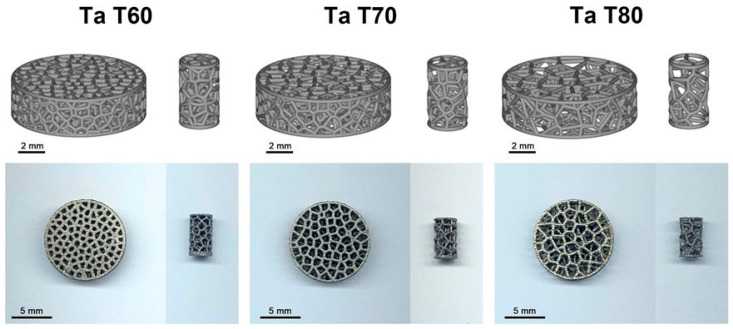
Three porous tantalum scaffolds with different porosities. (Adapted from [49]).

Metal materials struggle to form a tight bond with host tissue and have stress shielding issues, which impede osseointegration and lead to bone resorption [45]. Therefore, its porous structure has been optimized by researchers through design modifications, adjusting porosity to match the elastic modulus of human bone and simulate real mechanical conditions. Yu et al. [46] designed titanium scaffolds with the Primitive-Gyroid hybrid structure and simulated porosities of 50%, 60%, and 70% (i.e., PG50, PG60, PG70). PG50 was found to better match the mechanical properties and fluid transport requirements of human cortical bone, and was an ideal bone defect repair material with both stress-shielding resistance and osteointegration potential. Mahmoud et al. [47] investigated three types of Ti6Al4V cycloid bone implants. It was found that the apparent elastic modulus is influenced by geometric errors in the strut shape, and the fatigue strength depends on surface quality and internal pore distribution, which provides key design principles for balancing the manufacturing and performance of gradient-porous metallic lattices in bone implants. When CoCrMo plates were in contact with the cortical bone of the tibia in osteoporotic rabbits for 8 weeks, Zuchuat et al. [48] observed that the formation of new cortical bone was promoted locally, the number of pores within the cortex was reduced, and the Haversian system was reconstructed, but the effect was confined to the implant interface. This alloy was demonstrated to be a potential biomaterial for improving the fixation between damaged bone and implants. Jiao et al. [49] prepared tantalum scaffold beams with 60%, 70%, and 80% porosity via LPBF, as shown in Figure 5. In vitro and in vivo experiments confirmed that the 70% porosity scaffold exhibits both excellent osteogenic activity and osseointegration, and a direct basis for optimizing tantalum implant parameters was provided for clinical use. Soro et al. [50] combined the Ti-25Ta alloy with three TPMS lattices of gyroid, diamond, and Schwartz structures featuring 60% porosity. Following single-step LPBF fabrication, the designed implant-grade scaffold was prepared, and it had an equivalent strength to modulus ratio, higher ductility, and no cytotoxicity compared to Ti-6Al-4V. An ideal bone implant should not only possess mechanical properties similar to natural bone but also exhibit bioactivity, corrosion resistance, antibacterial properties, and osteoinductivity [51]. Therefore, the surface of materials is modified to impart advanced properties they originally lacked through methods such as coating, porosity enhancement, or loading with bioactive molecules or antibiotics, which is a major direction in the current evolution and development of metallic materials.

**Figure 6:**
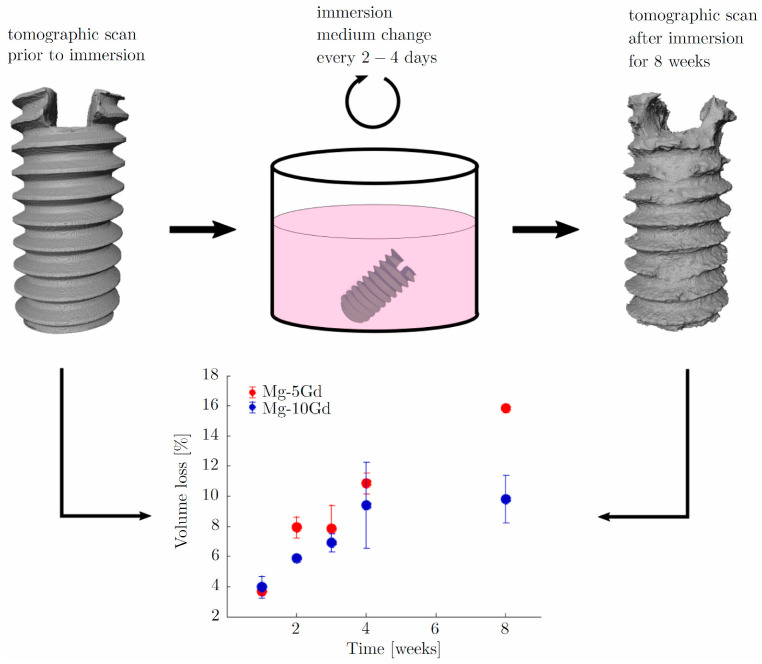
Schematic diagram of the experiment conducted. (Reproduced from [58] with permission).

Another major direction in the evolution and development of metallic materials is biodegradable metals. Biologically inert materials exhibit excellent stability and serve as physical scaffolds within defective bone tissue. However, they cannot degrade during the natural bone repair process, thereby impeding healing rates and causing inflammation or even cancerous changes in severe cases [52]. Therefore, the advent of biodegradable metals has replaced inert metals as temporary substitutes for defective bone, gradually dissolving as the bone heals. At present, biodegradable metals primarily include Mg and Mg -based alloys, iron (Fe) and Fe -based alloys, zinc (Zn) and Zn -based alloys [53]. Due to their excellent biocompatibility and mechanical properties, biodegradable metallic materials have become a hot research area in biomaterials. Through processes such as purification, alloying, and surface coating, the degradation rate can be controlled, and the antimicrobial properties, corrosion resistance, and osteogenic performance are enhanced [54]. Li et al. [55] designed a biodegradable bone implant (Mg-0.2Si-1.0Ca). Mg^2+^ was rapidly released during the early implantation phase to promote the bone immune microenvironment, and a biomimetic calcification interface based on MgO and Mg(OH)_2_ was self-assembled. Thus, dynamic regulation was released, the excessive corrosion was suppressed, and the precise coordination between degradation and bone regeneration was achieved. Morris [56] designed a novel implant prepared from magnesium and phosphate, and its fixation method. The implant was composed of a frame that could be inserted between adjacent vertebrae and an internal lattice structure, providing both initial stability to the vertebrae and space for bone ingrowth. Through material-structure-function integration, Zhao et al. [57] developed a biodegradable zinc-based implant, and its divalent ions were continuously released via the TPMS structure. Its mechanical properties were enhanced, and osteogenesis, immunomodulation, and antibacterial functions were achieved, providing a precisely tailored clinical translation solution for weight-bearing bone defects. Hermann et al. [58] proposed and validated a multiscale computational framework featuring sequential coupling of periodic dynamics and finite element methods. This framework was demonstrated to quantitatively reproduce the corrosion evolution of Mg-5Gd and Mg-10Gd alloy screws over several weeks, as shown in Figure 6. This mechanistic approach has been further advanced by the same group through the development of a nonlocal Nernst-Planck-Poisson system, which provides a more rigorous foundation for modeling the complex interplay of ion transport and electrochemistry during Mg corrosion [59]. A key challenge in the field is correlating accelerated in vitro tests with in vivo performance. The computational modeling proposed by Al Baraghtheh et al. [60] was used to quantify and coordinate the differences between the in vivo and in vitro degradation rates of Mg-xGd implants. To provide a precise mechanism and computational model for the degradation of Mg -based implants, the highest possible resolution was used to evaluate the nano-porosity and porous network of their degradation layer [61]. Degradation of Mg implants has been shown to exist with inherent regulatory mechanisms that can promote angiogenesis and reduce fibrosis [62]. These works provide a foundation for the development and clinical translation of biodegradable Mg alloys, helping to predict their in vivo performance and immunomodulatory behavior.

### 3.2. Polymer Materials

Polymer materials play an indispensable role in the field of bone implants. Compared to metallic materials, they typically exhibit excellent toughness, impact resistance, and low elastic modulus, significantly expanding the range of orthopedic treatment options. Based on whether they can be degraded and absorbed by the human body, they can be divided into two major categories: non-biodegradable polymeric materials and biodegradable polymeric materials.

**Figure 7:**
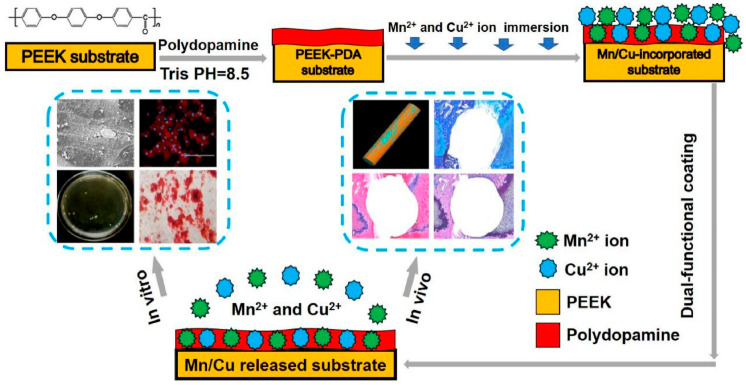
Schematic of the preparation of Mn/Cu co-deposition on PEEK using a PDA chemical surface modification and enhanced osseointegration while exerting excellent antibacterial activity in vitro and in vivo. (Adapted from [65]).

Non-biodegradable polymeric materials primarily include polyether ether ketone (PEEK) and ultra-high molecular weight polyethylene (UHMWPE). PEEK is a high-performance specialty engineering plastic characterized by outstanding mechanical properties, fatigue resistance, chemical corrosion resistance, and radiation permeability, which can effectively reduce the stress shielding effect [63]. UHMWPE exhibits exceptional wear resistance, impact resistance, chemical inertness, and a low coefficient of friction, and it is commonly used in joint replacements [64]. Non-biodegradable polymeric materials exhibit biological inertness, with surfaces that are unfavorable for bone cell adhesion and growth. They lack osteoconductivity or osteoinductivity and cannot form chemical bonds with bone tissue. Current research focuses on imparting bioactive properties and promoting osseointegration. One approach involves physically or chemically modifying the surface, and the other involves combining it with bioactive materials to prepare composite materials. Through the method of poly-dopamine (PDA)-manganese/copper (Mn/Cu) bimetallic ion co-deposition, Wang et al. [65] performed a simple surface modification on PEEK implants, which were conferred broad-spectrum antibacterial and osteoconductive functions. Their clinical lifespan was extended in infected bone defect environments, as shown in Figure 7. Oladapo et al. [66] fabricated PEEK-HAp bio-composites through FDM printing and surface microporation treatment. While maintaining mechanical stability, it is endowed with surface bioactivity, soft tissue ingrowth, cell proliferation, and osteogenic function are promoted, thus transforming inert PEEK into an osteo-soft tissue implant capable of integration. Kong et al. [67] employed hot-press nanoimprinting technology to construct precisely controllable nanoscale topographies on the surface of PEEK implants. Through purely physical morphological modification, the osseointegration capability is significantly enhanced while preserving the material’s inherent chemical properties, achieving both biocompatibility and clinical applicability. Aksu et al. [68] presented an improved implant made of PEEK for covering large-area sternal defects, the core was a lattice structure containing numerous ring-shaped units. The annular unit with through-holes could be flexibly secured to bones such as ribs, and the entire implant had good flexibility. Inverardi et al. [69] utilized UHMWPE bearing surfaces as platforms to load analgesics and antibiotics. By controlling the morphology-mechanical properties through process optimization, anti-Staphylococcus aureus activity and local analgesia were achieved, providing a novel strategy for multifunctional anti-infective implants. Li et al. [70] interlocked HA-modified UHMWPE with a PEEK scaffold to create a boundary lubrication layer capable of sustained HA release, reducing the coefficient of friction to 0.041. This approach combines excellent biocompatibility with early osseointegration, offering a highly wear-resistant UHMWPE-PEEK composite solution for joint replacement. The biomechanical impact of these polymers extends beyond their intrinsic properties to their interaction with the host bone. A critical consideration is the relationship between implant stiffness, interfacial micromotion, and stress shielding. This underscores that the biological performance of these materials is governed not only by their surface chemistry but also by the mechanical environment they create. For example, the parameters of the porous structure were altered to adjust stiffness and reduce stress shielding, and it was confirmed in animal experiments that it can promote bone healing [71]. Additionally, computational frameworks can be designed based on principles of mechanobiology (such as bending loads), which can provide an in-depth understanding of the influence of mechanical factors on the lumbar fusion process [72].

**Figure 8:**
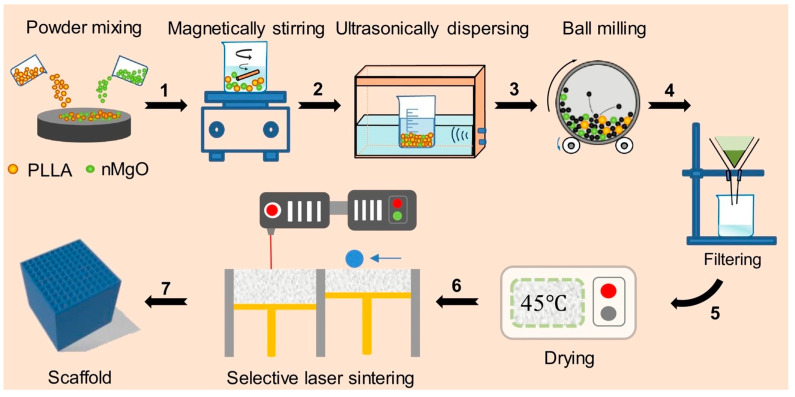
Fabrication process of the bone scaffold. (Reproduced from [74] with permission).

Biodegradable polymer materials are an important direction for the future development of bone implants [73]. After fulfilling their temporary support and fixation functions, these materials can be gradually degraded and absorbed by the human body, and are completely replaced by newly formed bone tissue, thereby eliminating the need for secondary surgical removal []. The regulation of their mechanical properties, degradation behavior, and surface functionalization remains a core challenge in current research. Through composite engineering and structural design, the performance of polymer-based implants continues to be enhanced. Shuai et al. [74] incorporated nano-magnesium oxide (nMgO) into a left-handed polylactic acid (PLLA) scaffold to construct a bone repair scaffold with an anti-inflammatory microenvironment, as shown in Figure 8. Under the combined effects of both factors, crystallization speed is accelerated, strength is increased by 38%, modulus is enhanced by 24%, and acidic degradation products are neutralized. Omigbodun et al. [75] found that increasing the HAP content in PLA/cHAP composite scaffolds enhances compressive and tensile strength. Its mechanical properties could be accurately predicted by constructing machine learning regression algorithms. In addition to the data-driven characteristic prediction discussed above, the interaction between mechanics and biology had been investigated by simulating scaffold structures and loading conditions using computer modeling techniques, indicating that the mechanical environment of the scaffold influences bone regeneration [76]. By adjusting the extrusion width and nozzle temperature, Bakhtiari et al. [77] enhanced the compressive strength and fatigue life of PLA scaffolds while maintaining a 60% porosity, the hysteresis temperature rise remained below the glass transition temperature, and the potential of PLA as a fatigue-resistant bone scaffold material was validated. Hari Raj et al. [78] optimized the design of PLA biodegradable screws and modified them with a TiO_2_-ZrO_2_ nanocomposite coating. Through various characterization techniques and in vitro experiments, the coating was confirmed to enhance surface bioactivity and osseointegration, thereby providing a basis for developing high-strength, high-compatibility absorbable orthopedic implants.

### 3.3. Ceramic Materials

Due to their exceptional biocompatibility, outstanding wear resistance, and composition similar to bone tissue, ceramic materials are highly favored in the field of bone implants and occupy a pivotal position. Based on their chemical activity in biological environments, they can be classified into biocompatible ceramics and bioactive ceramics [79].

**Figure 9:**
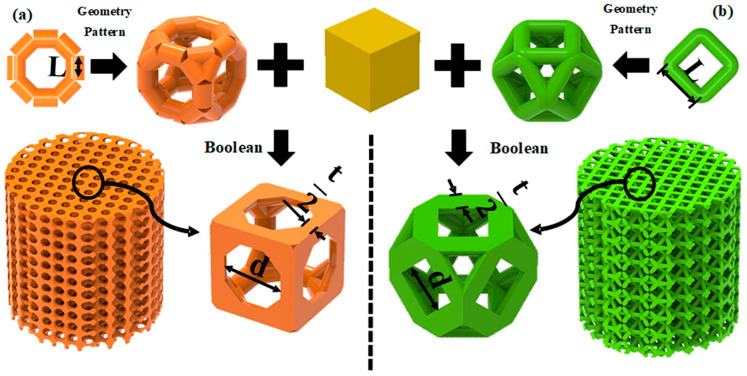
Modeling diagram of two types of porous prostheses. (**a**) Octagonal structure; (**b**) Rhombic structure. (Reproduced from [82] with permission).

Biologically inert ceramics have stable chemical properties and are not easily reactive in biological bodies, such as alumina, zirconia, silicon nitride, etc. Alumina and zirconia have extremely high hardness, hydrophilicity and corrosion resistance, and are the first ceramic materials to be applied to artificial joints. But single materials are prone to fracture and failure. To enhance the overall stiffness and toughness of the material, biphasic ceramics such as alumina toughened zirconia (ATZ) have been developed [80]. Silicon nitride, with non-cellular toxicity, high toughness, and high wear resistance, has been proven to be usable as a biomaterial and has great potential in the field of bone implants [81]. Lei et al. [82] prepared octagonal and rhombic Al_2_O_3_ scaffolds with varying porosities, as shown in Figure 9. Octagonal scaffolds with 0.566 mm pore size and rhombic scaffolds with 1.0 mm pore size were demonstrated to possess suitable mechanical properties, permeability. Malmström [83] designed a customized inert ceramic implant for bone repair. Its inner surface features lamellar or beam-like structures that precisely conform to the bone’s shape. Secured with fixation screws, it formed a rigid protective layer that shielded against external forces, which provided a stable mechanical environment for bone healing and optimized treatment outcomes.

**Figure 10:**
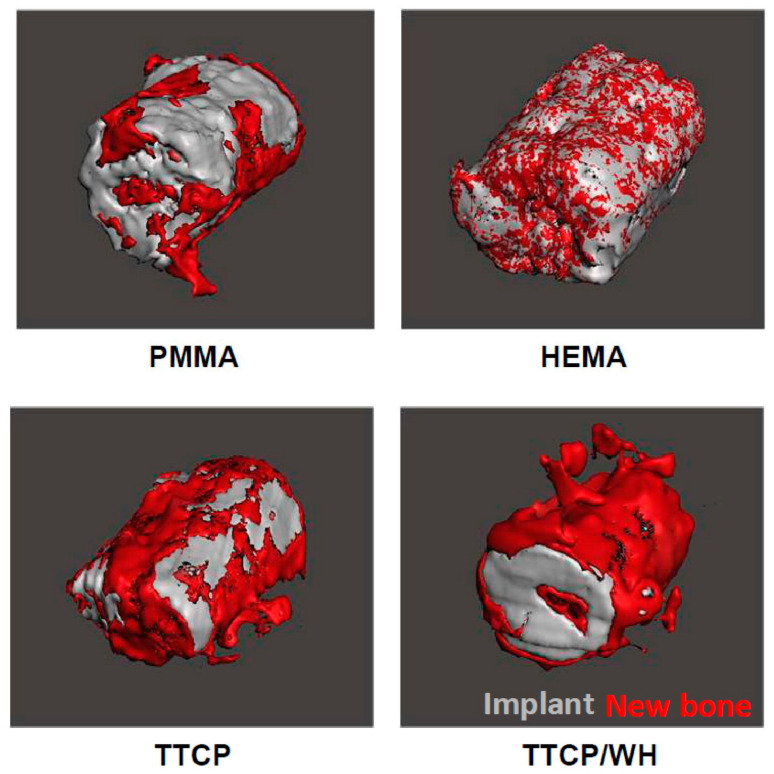
New bone formation images around implanted scaffolds. (Reproduced from [88] with permission).

Bioactive ceramics are mainly calcium phosphate ceramics (CPC) and bioactive glass (BAG), which are similar to the main components of the inorganic matter of human bone tissue. Therefore, it has excellent biocompatibility and osteoconductivity and can chemically react with the surrounding bone tissue to achieve osseointegration. Calcium phosphate ceramics can be further categorized into hydroxyapatite (HA/HAp), tricalcium phosphate (β-TCP, α-TCP), and tetracalcium phosphate [84]. HA is rarely used alone in load-bearing areas, and it is mainly used as a coating for metallic implants and as a bone defect filler to promote bone growth and achieve biological fixation. As a high-temperature phase of calcium phosphate, β-Tricalcium phosphate (β-TCP) has good degradability and biocompatibility. Calcium phosphate mixture is a new direction in material development, such as biphasic calcium phosphate (BCP). By adjusting the ratio of HA and β-TCP, the absorption rate and bioactivity of BCP can be regulated, promoting osteogenic differentiation of cells [85]. In addition to bioactive ceramics, functional ceramics (such as piezoelectric ceramics) are another important branch of advanced ceramics. When encountering external stimuli, piezoelectric ceramics (barium titanate, magnesium silicate (barium titanate, magnesium silicate, etc.) generate surface charges to promote cell proliferation, providing a new solution for bone repair [86]. Mahmud et al. [87] modified ZK41 magnesium alloy using anodic oxidation and HA coating strategies, its Vickers hardness doubled, and the corrosion rate was reduced to 0.018 mm/year. Degradation was delayed while high cellular activity was maintained, demonstrating HA-ZK41 as a viable material for degradable implants. Liu et al. [88] incorporated tricalcium phosphate (TTCP) and Whitlockite (WH) into polymethyl methacrylate (PMMA) to develop functional bone cement. The tests demonstrated that the working time was shortened and the modulus was reduced. Its bone conduction and integration were proven to be superior to pure PMMA, enabling synergistic effects of support and bone formation, as shown in Figure 10. Roslinda et al. [89] designed a composite bone regeneration scaffold centered on a porous structure composed of TCP and HA, with pore sizes ranging from 100 to 5000 μm. By cross-linking biocompatible polymers within the ceramic network, bioactive components were firmly anchored within the scaffold, thereby synergistically promoting bone repair and growth. Huri et al. [90] designed a polymer/β-TCP composite scaffold that supports cell infiltration and vascularization through its unique angled layered structure, and employed deep coating with HA solution to enhance growth factor delivery efficiency. Its unique porous structure and surface microcrack treatment enhanced bioactivity and promoted bone tissue regeneration.

### 3.4. Considerations for Material Selection

**Figure 11:**
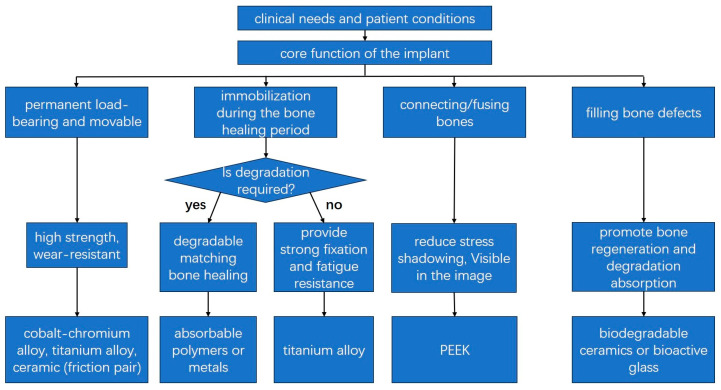
Decision-making process for bone implant materials.

Based on the above introduction, metals, polymers, and ceramics all demonstrate excellent performance as bone implants and can be tailored to different medical scenarios. The advantages, disadvantages, and comparative selection criteria for each material are summarized in Table 2. Selecting materials for bone implants is a complex multi-objective decision-making process that requires surgeons and engineers to strike an optimal balance among competing factors based on the patient’s clinical needs and material properties. For instance, metal is the preferred choice for areas requiring mechanical support, such as bone plates. Inert ceramics or highly crosslinked polyethylene are preferred for joint heads requiring wear-resistant surfaces. Degradable ceramics or calcium sulfate bone cement are preferred for bone defects requiring bone growth. The decision-making process for bone implant materials is shown in Figure 11.

The selection of bone implant materials is the result of a comprehensive evaluation of all these factors, including the intrinsic properties of the material (mechanical, biological characteristics), the patient’s clinical needs (load-bearing requirements, bone quality), the expected function of the implant (providing support, filling defects), and the operability of the surgery. However, single materials face significant limitations in practical applications. Advances in materials science have focused on enhancing material properties through surface modification, porous structure design, and composite materials, thereby combining the advantages of different materials to meet complex clinical demands. Chen et al. [91] loaded gentamicin-loaded gelatin into a titanium alloy scaffold. Escherichia coli and Staphylococcus aureus were effectively inhibited at a drug loading of 8 mg/mL, and the bone implant composite material was constructed, combining mechanical support, sustained drug release, and biocompatibility. Hussain et al. [92] prepared a tannin-modified nanoscale composite of Fe-HA-polycaprolactone (PCL). Its excellent biocompatibility, antioxidant activity, and osteogenic properties were demonstrated through in vivo and in vitro experiments, and it could be used as an efficient interfacial scaffold to promote osseointegration. Ouldyerou et al. [93] systematically evaluated the impact of six designs of Ti-PEEK composite implants on marginal cortical bone stress shielding. They found that under conditions without marginal bone loss, the composite structure featuring an outer PEEK layer encapsulating a Ti core reduced implant-bone stiffness mismatch and diminished the stress shielding effect, and the mechanical performance was superior to that of conventional dense titanium implants. Sheth et al. [94] designed a bioactive filamentous structure based on composite materials, the synthetic bone graft particles (such as BAG) were attached to the filamentous structure, and the particles were fixed and formed a coated scaffold structure through polymer precipitation (such as PCL). It could be used to enhance surgical sutures, endowing them with the ability to promote tissue regeneration and osseointegration. Wang et al. [95] proposed a Ti/Ta porous scaffold by coating a porous Ta layer onto the periphery of a porous Ti framework. This design combined the high specific strength and low modulus properties of Ti alloys with the excellent osteogenic activity of Ta metal. Its dual-material heterogeneous pore structure significantly enhanced osseointegration capability while maintaining mechanical performance.

## 4. Manufacture Method for Bone Implants Based on Additive Manufacturing

To enhance the therapeutic efficacy of orthopedic surgery and extend the service life of bone implants, artificial bone implants should match the external contour morphology of the patient’s native bone while featuring an internal porous gradient structure. However, traditional casting techniques struggle to achieve this objective. The advent of additive manufacturing technology has overcome this challenge. This technique involves the gradual layering of materials to create three-dimensional solid parts, and complex shapes and porous materials can be fabricated to meet patients’ individualized needs [96]. At present, the primary additive manufacturing technologies used for bone implant materials include Selective Laser Melting (SLM), Electron Beam Melting (EBM), Fused Deposition Modeling (FDM), Binder Jetting (BJ), and Stereolithography (SLA).

### 4.1. Powder Bed Fusion

Powder Bed Fusion (PBF) is one type of additive manufacturing. By spreading particles of materials such as metals, ceramics, and polymers onto a platform surface to form a powder bed, the powdered material is fused layer by layer using thermal energy, thereby producing efficient, precision parts. Based on the heat source and temperature range, these processes can be further categorized into four distinct types: Selective Laser Melting (SLM), Selective Laser Sintering (SLS), Electron Beam Melting (EBM), Multi-Jet Fusion (MJF). Among these, SLM and EBM are typically used for printing metal parts due to their inherent processing characteristics, so they are widely applied in the manufacturing of metal bone implants.

#### 4.1.1. Selective Laser Melting

**Figure 12:**
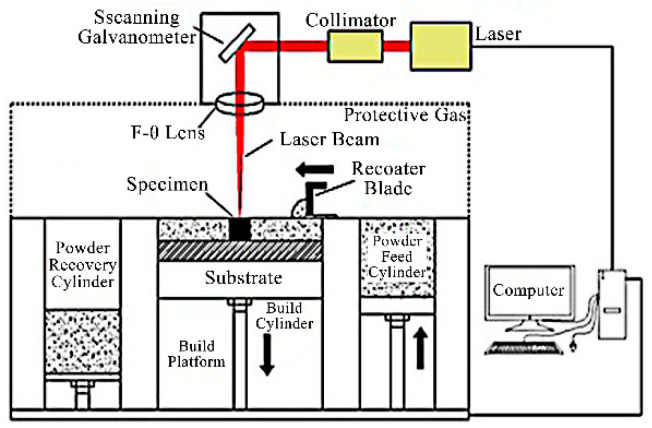
Schematic Diagram of SLM Forming Principle.

SLM technology offers high precision and high freedom of form. It can process parts with arbitrarily complex structures and can even manufacture porous implants with variable porosity. The microstructure and mechanical properties of this implant better match those of human bone, thus offering significant application potential in the field of personalized biomedical bone implants. The forming principle of SLM technology is shown in Figure 12.

**Figure 13:**
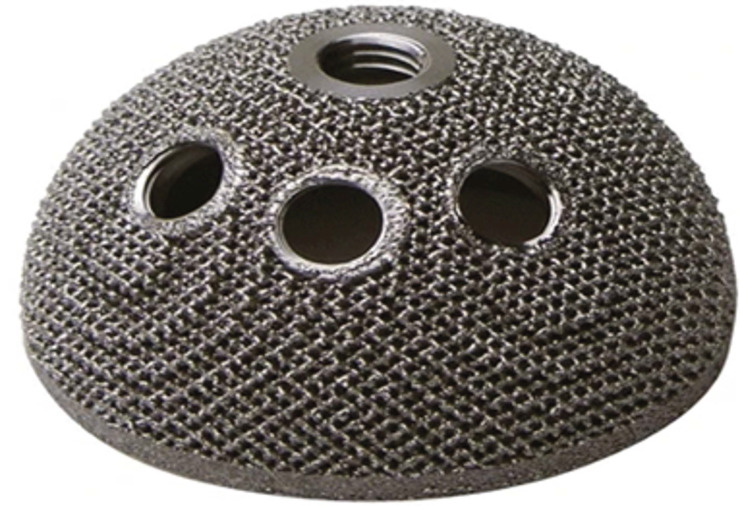
Ti-alloy porous acetabular cup implant. (Adapted from [97]).

Research on SLM in the field of bone implants is highly active, focusing on innovative optimization of porous structures, optimization of process parameters, and personalized customization. SLM technology enables the fabrication of metal implants with complex porous structures. These structures not only deliver superior mechanical properties and biocompatibility but also significantly promote inward bone tissue growth and integration. Perticarini et al. [97] utilized SLM technology to fabricate a porous titanium alloy acetabular cup implant, as illustrated in Figure 13. This implant possessed a porous structure with excellent osseointegration capability and was applied to the human body to effectively promote bone ingrowth, cell adhesion, and growth. Zheng et al. [98] utilized SLM technology and titanium alloy to fabricate a skull base-temporomandibular joint prosthesis, which exhibits excellent biocompatibility and mechanical properties, and it was mainly used for reconstructive repair following surgery for skull base and temporomandibular joint pathologies. Ataee et al. [99] utilized SLM and EBM technologies to fabricate multi-group gyroid bone scaffolds from CP-Ti and Ti64 materials, respectively. In vitro culture experiments were conducted to compare the two scaffolds. Results indicated that the bone scaffolds produced via SLM demonstrated superior biocompatibility, with greater surface area correlating to enhanced biocompatibility. Wauthle et al. [100] produced porous femoral implants with identical pore structures of tantalum and titanium alloys using SLM technology, and implanted them into two rats, respectively. After 12 weeks, substantial inward bone growth was observed in both rats, with regenerated bone exhibiting good quality. Compared to titanium alloy implants with identical pore structures, pure tantalum implants demonstrated superior bone conduction properties. Barba et al. [101] investigated the design flexibility of four implant structures of Gyroid, IWP, Diamond, and Neovius. The four implant types were fabricated using SLM technology and subjected to testing. Results indicated that both Neovius and IWP structures provided suitable bone growth regions, with the Neovius structure demonstrating the highest design flexibility. Jannesar et al. [102] manufactured porous Ti implants using SLM technology, which can achieve structural integrity through a porous surface layer several millimeters thick. Their pore structure mimics natural bone tissue with precisely controllable pore parameters, making it suitable for producing customized implants in orthopedic and dental applications.

In summary, the implants manufactured by SLM demonstrate outstanding performance in terms of bone conductivity and cell compatibility. By regulating the microscopic topography, the bone ingrowth space and design flexibility can be further optimized, providing a reliable solution for personalized bone repair. However, inherent residual stresses in the process can damage the fatigue life of porous structures under cyclic physiological loads, and the generation of wear particles in articulated components can affect bone integration [103]. Solving these problems requires interdisciplinary knowledge, such as materials science and biomechanics, to reduce residual stress and optimize surface roughness.

#### 4.1.2. Electron Beam Melting

**Figure 14:**
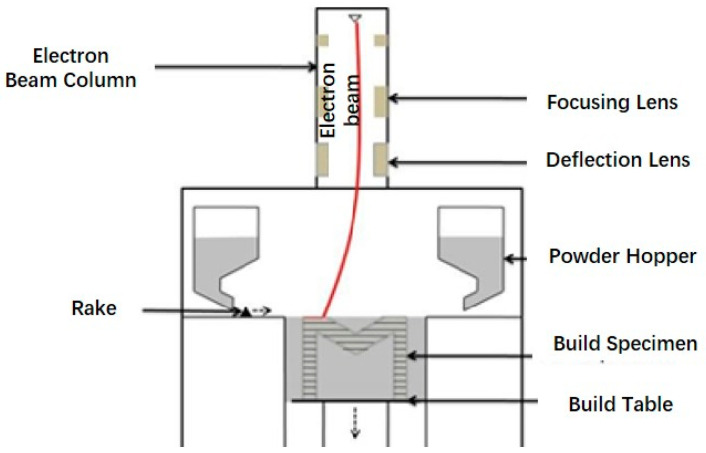
Schematic diagram of the EBM process. (Reproduced from [104] with permission).

As the same category of powder bed additive manufacturing, EBM shares a highly similar process flow with SLM, as shown in Figure 14 [104]. The difference lies in the fact that EBM uses an electron beam rather than a laser beam as the heat source. High-power electron beams operate at extremely high speeds and can function in high-temperature environments, and the residual stress levels in parts prepared after annealing are significantly lower than those produced by SLM, which can be used to process high-performance materials [105]. Compared to SLM, EBM demonstrates superior comprehensive performance in porous titanium alloy stents and is currently one of the mainstream approaches for fabricating orthopedic implants [106]. Wang et al. [107] demonstrated that Ti6Al4V regular porous scaffolds constructed via EBM significantly promoted the adhesion, proliferation, and osteogenic differentiation of human bone marrow mesenchymal stem cells in vitro. In rabbit femoral condyle defects, these scaffolds yielded superior new bone formation compared to the PT-SLM group, and the mechanical properties and osseointegration were maintained without excessive removal of the partially melted powder. Watanabe et al. [108] employed EBM to fabricate high-porosity Ti-6Al-4V acetabular cups, with half of them modified by nHA coating. In experiments with beagle dog femoral shaft grafts, it was found that early pull-out force increased and the bone tissue grew into deep pores within 4–12 weeks. nHA was demonstrated to effectively inhibit the occurrence of radiolucent lines around porous cups prepared using EBM. Based on EBM and Ti-6Al-4V, Ren et al. [109] constructed a microgroove-nanotube hierarchical morphology in the primary surface by ultrasonic acid etching and anodic oxidation. After 8 weeks, BV/TV was raised to 43.4%, the bone contact area increased, and EBM combined with micro/nano modification was proven to overcome the bottleneck of titanium’s biological inertness, accelerating osseointegration. Bandekhoda et al. [110] found that Ti-6Al-4V prepared by EBM exhibits no α’-martensite and rich β-phase, and its corrosion resistance and bioactivity in SBF were superior to those of SLM-prepared specimens. It was also found that while the polished state enhances corrosion resistance, the high roughness of the finished samples increases surface activity by boosting OH^−^ group content. Szymczyk-Ziółkowska et al. [111] subjected Ti-6Al-4V ELI alloy formed by EBM to HIP post-treatment. Through high-temperature diffusion, microdefects were eliminated, the microstructure coarsened, the plasticity enhanced, and the corrosion resistance significantly improved compared to the untreated state, providing a key strategy for corrosion control in personalized medical implants. Gatto et al. [112] utilized EBM technology and a hybrid powder blend to fabricate diamond-like (DO) and rhombic dodecahedral (RD) scaffolds. Their low-roughness surfaces directly promoted hMSC proliferation, and the PCL/HA coating further enhanced biocompatibility. Mechanical properties aligned with literature data for novel powders confirmed that EBM enables low-cost, scalable production of bone scaffolds without compromising quality. Wu et al. [113] proposed a personalized titanium metatarsal implant manufacturing method based on EBM technology. Through drug coating and anodic oxidation treatment, the implant’s mechanical adaptability, corrosion resistance, and osseointegration capability were enhanced, resulting in improved initial stability and accelerated rehabilitation progress.

Research indicates that EBM can produce scaffolds with both excellent mechanical properties and highly interconnected porous structures, providing an ideal environment for bone cell ingrowth and nutrient delivery. Furthermore, unique metallurgical processes can endow the material with superior corrosion resistance and bioactivity. The scaffolds prepared via EBM provide an excellent substrate for subsequent surface modification, accelerating osseointegration and suppressing adverse tissue reactions. They hold great promise in the field of high-performance and personalized biomedical implants.

### 4.2. Fused Deposition Modeling

**Figure 15:**
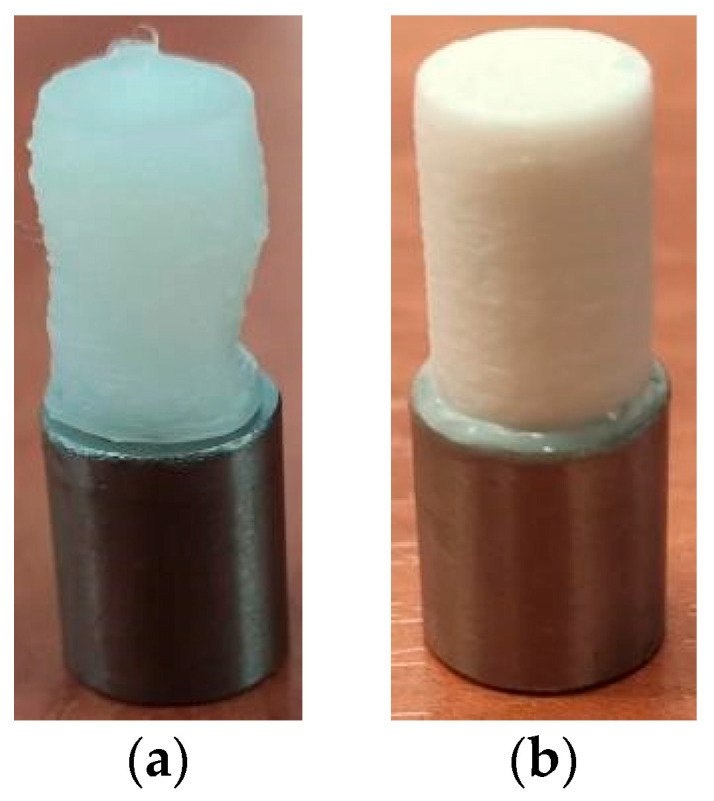
Obtained prints. (**a**) deformed PA overprint on a sample that was not cooled down after three layers were applied; (**b**) correctly printed PLA overprint. (Reproduced from [118] with permission).

FDM is currently one of the most popular and widely used 3D printing technologies. The principle involves melting thermoplastic filament through heating, then extruding it layer by layer, like squeezing toothpaste through a nozzle to build up the object, and finally cooling and solidifying it to form a three-dimensional solid. Therefore, FDM technology is commonly used in the field of bone implants to print bioactive polymers such as polycaprolactone (PCL), polylactic acid (PLA), and polylactic-polyhydroxyacetic acid copolymer (PLGA). Based on its core advantages, such as controllable porous structures and relatively low cost, researchers have explored its application in manufacturing bone repair scaffolds, developing novel composite materials, and creating drug delivery systems. Oladapo et al. [114] employed FDM printing to fabricate PEEK-rGO-cHAp homogeneous porous hip implants. Through synergistic design of lattice structure, porosity, and surface functionalization, they achieved bone-like elastic modulus, controllable microporosity, and excellent biocompatibility, and they could enhance cell infiltration and osseointegration. Bassand et al. [115] compared the effects of two 3D printing techniques on ibuprofen-PLGA implants, confirming that the FDM-prepared mesh structure exhibits high porosity due to its open architecture formed by continuous straight filaments. It achieves substantial drug release within 4 days, accompanied by rapid convective mass transport, making it suitable for biodegradable implants requiring a rapid onset of action. Wojnicz et al. [116] proposed a mathematically driven bone implant design methodology. Through finite element optimization of nine types of periodic scaffolds, they achieved effective Young’s moduli matching lumbar trabecular bone in both Ti-6Al-4V and ABS materials. Seven types of ABS brackets were successfully printed using unsupported FDM technology, with experimental modulus values matching numerical predictions. Oladapo et al. [117] successfully fabricated PEEK-cHAp composite scaffolds using FDM, achieving dense interlayer bonding and high dimensional accuracy. They found that 10–20 wt% cHAp significantly enhanced tensile strength and stiffness while maintaining cell compatibility, offering a rapid, low-cost customized solution for femoral defects. Grygier et al. [118] demonstrated the feasibility of depositing polylactic acid (PLA) and polyamide (PA) onto Ti6Al4V surfaces via FDM, as shown in Figure 15. However, the interfacial tensile/shear strength proved insufficient and lacked reproducibility, and the process or material system requires further optimization to achieve metal-polymer integrated implantable prostheses.

In summary, FDM technology demonstrates its unique value and flexibility in the field of preparing bone repair scaffolds and implants. Its exceptional material adaptability enables the processing of polymers, bioceramics, and their composites to meet diverse mechanical and biological requirements. Through structural design optimization and porosity regulation, it can precisely match the elastic modulus of bone tissue and achieve programmable control over drug release rates, cellular infiltration, and nutrient delivery functions. However, this technology still faces challenges in achieving interfacial bonding during metal-polymer integrated molding, which requires further process innovation.

### 4.3. Binder Jetting

**Figure 16:**
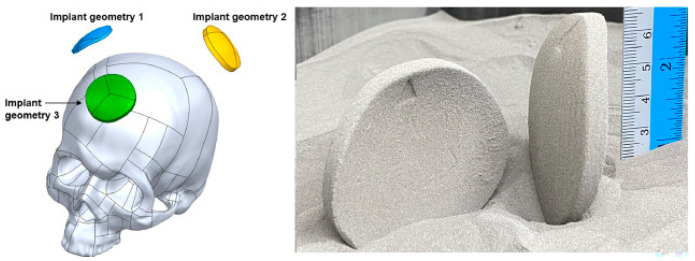
BJ printed spherical cap-shaped Mg implants. (Reproduced from [120] with permission).

BJ is essentially another additive manufacturing technology based on powder beds. The difference lies in the fact that it fixes powdered materials using an inkjet printhead and adhesive, rather than employing laser melting. BJ exhibits excellent material compatibility and can be used with polymeric materials, metallic materials, and ceramic materials. This enables it to meet the demand for customized implants in diverse mechanical environments, demonstrating particularly significant value in the field of craniofacial surgery. However, its post-processing is complex, and the printed part must be sintered at high temperatures to obtain the final performance. This process can lead to distortion of parts and requires precise process control to ensure dimensional accuracy. Huang et al. [119] subjected HA scaffolds fabricated via BJ technology to 5 wt% citric acid post-treatment at room temperature. The PVA membrane thickness was increased to 320%, and the compressive strength and modulus were enhanced to 840.7% and 1571.3%, respectively, meeting the criteria for cancellous bone and providing a straightforward approach for strengthening brittle bioceramics with low residual stress. Salehi et al. [120] established an end-to-end digital chain integrating BJ with automated dry post-processing. Through sintering on a graphite substrate, porous Mg implants with spherical cap shapes achieved 15.2% free shrinkage and reached 87% relative density. Subsequent automated dry finishing reduced average roughness to <1.3 μm, enabling precision manufacturing of customized porous magnesium scaffolds, as shown in Figure 16. Xie et al. [121] fabricated Ti6Al4V uniform and gradient structures (U-GLS, G-GLS) via BJ molding. While maintaining dimensional accuracy within ±2%, the U-GLS exhibited higher yield strength and energy absorption due to its 45° shear zone, the G-GLS exhibited layer-by-layer collapse and delivered stable energy absorption efficiency, providing a basis for performance design of BJ-TPMS structures in bone implants and energy-absorbing components. Salehi et al. [122] employed BJ printing and sintering to fabricate Mg–Zn–Zr scaffolds in a single step. The alloy properties were precisely tuned, achieving tensile/yield strengths of 130/100 MPa and a modulus of 21.5 GPa. This approach enabled mechanical-biological matching and established a process foundation for digitally customized biodegradable magnesium alloys. Through gray relational analysis, Sahu et al. [123] optimized process parameters such as layer thickness, orientation, and saturation in BJ. The synergistic improvement in compressive strength, porosity, and dimensional accuracy of ceramic scaffolds was achieved, the experimental deviation was verified to be <4%, and the optimal window for preparing high-precision porous bone scaffolds via this process was established.

BJ technology enables the fabrication of complex structures with exceptional dimensional accuracy across diverse materials, ranging from ceramics and metals to biodegradable active materials. However, critical process challenges must be overcome, particularly for materials like magnesium and ceramics. These include precise dimensional control to compensate for part shrinkage during sintering, and the mitigation of sintering defects (e.g., microcracks, porosity) that can severely compromise the mechanical reliability of the final implant [124]. Through innovative post-processing techniques, inherent challenges such as low strength in brittle materials, difficulty in controlling metal shrinkage, and high surface roughness have been overcome, enabling the realization of both mechanical properties and biological functionality in the scaffold.

### 4.4. Stereolithography

**Figure 17:**
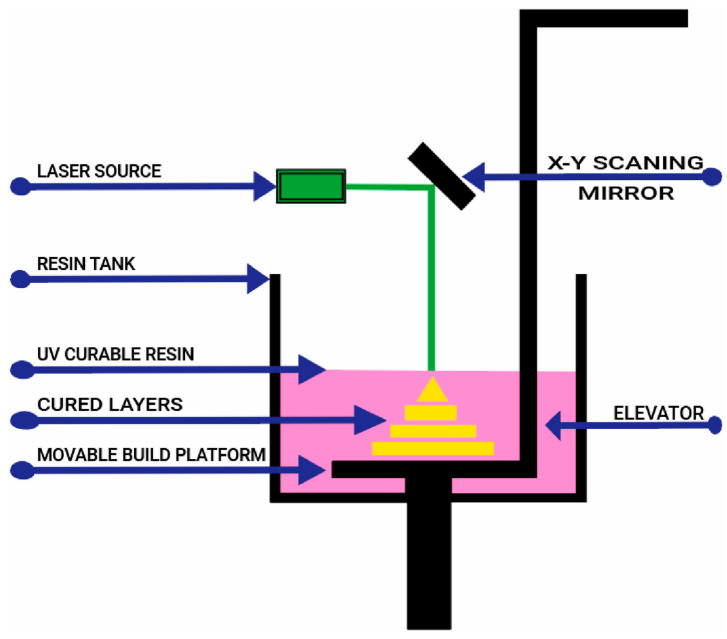
Stereolithography 3D printing. (Reproduced from [125] with permission).

**Figure 18:**
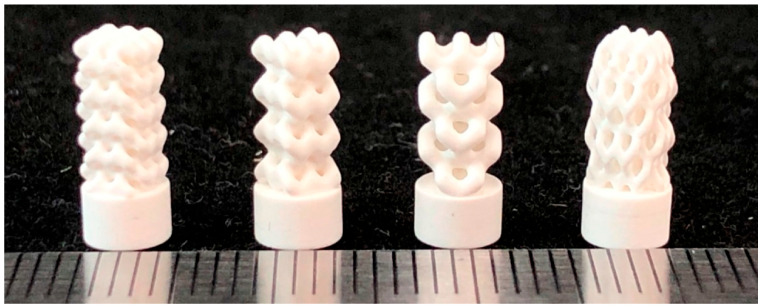
Four types of ceramic bone implants structures. (Reproduced from [126] with permission).

Ceramic materials generally have the dilemma of difficult cutting and forming, and are difficult to be prepared by traditional processes. By controlling specific wavelengths of ultraviolet light with computers, SLA technology can selectively cure flowing ceramic slurry, ultimately transforming it into a precision-structured high-performance ceramic, as shown in Figure 17 [125]. Therefore, SLA is one type of laser rapid prototyping technology, featuring excellent feature resolution and surface smoothness. Ceramic slurry mainly contains ceramic powder, photosensitive resin, and various additives (dispersants, photoinitiators, etc.). Stable materials and meticulous post-processing are key factors in manufacturing high-precision, complex-shaped ceramic implants. Safonov et al. [126] prepared alumia bone implants with customized porous structures using SLA technology, and four types of structures are shown in Figure 18. Wang et al. [127] developed a novel aqueous HA suspension for SLA technology, possessing good rheological properties and curing performance. By optimizing parameters such as surfactant concentration, solid loading, and particle size, ceramic components meeting the strength requirements of trabecular bone were successfully fabricated. Besides ceramics, SLA technology is also widely used for printing polymers and their composite materials. Bergmann et al. [128] fabricated customized bone implants composed of β-TCP and BAG, with good biodegradability and biocompatibility, for repairing defects in the maxillofacial and cranial bones. Kebede et al. [129] prepared HAp-dimethylacrylamide material with excellent mechanical properties via SLA, achieving a tensile strength of 30.2 MPa, which can be applied in the field of bone regeneration.

### 4.5. Comparison of Different Additive Manufacturing Technologies

In the field of orthopedic implant manufacturing, additive manufacturing technology has been widely used for its ability to accurately manufacture complex porous structures that are highly compatible with the patient’s anatomy. But there are numerous categories, each with its own emphasis, so that the functional requirements, mechanical load, biocompatibility, and cost-effectiveness of the implant must be considered comprehensively. For example, SLM and EBM, which represent metal melting technology, have become the mainstream and gold standard for permanent implant fabrication in load-bearing areas due to their ability to create parts that are completely dense and have excellent mechanical properties [130]. Among them, SLM is better in terms of precision, while EBM has an advantage in mass production due to its low stress and high speed. With its low cost and ease of operation, FDM technology has captured the market for preoperative planning models and surgical guides, while also making valuable inroads into the application of high-performance polymer implants. BJ offers exceptionally high printing speeds and the capability to fabricate complex porous structures, and it shows tremendous potential in manufacturing biomimetic porous scaffolds designed to promote bone ingrowth, particularly for repairs in non-weight-bearing or low-weight-bearing areas. SLA is one of the additive manufacturing technologies used to produce precise and complex components, advancing the manufacturing process of advanced bioceramics. The preceding section provides an in-depth examination of research on these five technologies in the field of bone implants, with a detailed comparison presented in Table 3.

## 5. Performance Evaluation of Personalized Bone Implants

The success of the bone implant depends on its performance in practical applications. This performance is determined by the interaction between the selected structural type, material system, and manufacturing method. For example, SLM and EBM technologies, which primarily use titanium and cobalt-chrome alloys, can produce implants with high mechanical strength and fatigue resistance, making them the benchmark for load-bearing applications in hip and knee joints. FDM technology can print biodegradable polymers such as PLA, thereby creating scaffolds with controllable degradation rates. It can also incorporate antibacterial agents, focusing on their bioactive effects in treating non-critical defects. BJ and SLA technologies utilize the potential of advanced ceramics (such as HA, β-TCP) and their composites. Implants with excellent bioactivity and osteoconductivity are manufactured for bone defects in craniofacial and dental regions. Therefore, key performance indicators such as mechanical properties, antibacterial efficacy, and osseointegration ability need to be evaluated. These performance characteristics are direct results of the choices made during the design, material, and manufacturing stages.

### 5.1. Mechanical Property

**Figure 19:**
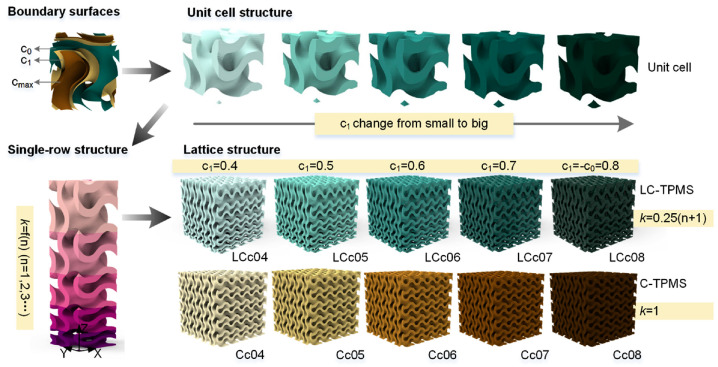
Design methods for LC-TPMS and C-TPMS lattice structures. (Reproduced from [135] with permission).

During daily activities, bones are subjected to many forms of loads such as compression, bending, shear, and tension, requiring the bone implant structure to be able to withstand the corresponding loads. Second, the modulus of elasticity of human bone does not match that of common implant materials and is prone to stress-masking effects, which can lead to implant loosening or even dislodgement [131]. Based on these problems, researchers have designed and optimized the topology of bone implant materials to better match the human skeleton. Therefore, the regulation of the mechanical properties of bone implants is an important direction of current research to modify and structurally optimize the materials to improve their use and delay their service life. Using the design approaches of equal-area thickness and volume, Wu et al. [132] fabricated porous titanium scaffolds with orthotropic cells arranged along a radial gradient. These scaffolds achieve higher strength while maintaining an elastic modulus matching that of tibial cortical-cancellous bone, and empirical formulas were established for predicting the mechanical properties of tibial implants. Huang et al. [133] fabricated Ti6Al4V auxetic and non-auxetic porous specimens via SLM and conducted fatigue tests within the yield strength range of 5–60%. Results showed that the non-auxetic structure exhibited significantly superior fatigue life compared to the auxetic structure. Further optimization of the auxetic unit cell topology was required to enhance fatigue strength while maintaining the mechanical advantage of a negative Poisson’s ratio. Chen et al. [134] conducted static simulations on three microporous units, discovering that adjusting their dimensions could regulate the elastic modulus of TC4 titanium alloy implants to match human bone ranges. Rod units provided high modulus and high flexural strength, column units covered a broad modulus range, and spherical units suited low modulus requirements. Zhang et al. [135] investigated the effects of porosity and pore size distribution on the performance of porous structures. They found that under equivalent porosity, linear-changing porous structures (LC-TPMS) exhibited higher yield strength, longer stable platforms, greater energy absorption, and higher damping ratios compared to constant-porosity porous structures (C-TPMS). Pore size gradient designs were demonstrated to significantly enhance performance without sacrificing porosity, with the design methodology illustrated in Figure 19. Cheng et al. [136] fabricated mandibular implants using a functional gradient TPMS structure, regulating mechanical properties through Z-axis gradient design. The top and bottom layers utilized high-strength units to bear the interlocking load, and the middle layer maintained high porosity to balance mechanical support with permeability requirements. This porous structure effectively conducted stress, reduced stress shielding, and achieved mechanical properties matching those of natural bone.

The core objective of current bone implant research is to achieve mechanical compatibility with human bone and to realize synergistic innovation in material selection, manufacturing processes, and structural design, thereby avoiding stress shielding effects and ensuring long-term stability. In the future, the fatigue behavior of novel structural concepts such as negative Poisson’s ratio will be thoroughly investigated, the interface strength of composite structures will be optimized, multi-scale hierarchical structural designs will be innovated, and reliable performance prediction models will be established. These are expected to represent key directions for further breakthroughs in overcoming the mechanical performance bottlenecks of bone implants.

### 5.2. Surface Antimicrobial Properties

**Figure 20:**
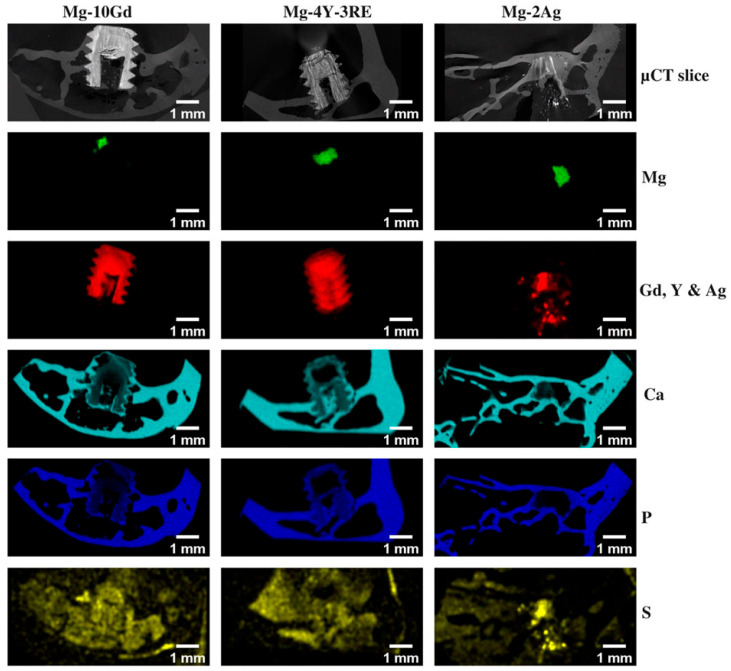
Exemplary µXRF maps of Mg-10Gd, Mg-4R-3RE and Mg-2Ag screws after 9 months of implantation. (Reproduced from [142] with permission).

The occurrence of bacterial infections in implants is an important issue in the field of orthopedic treatment. Since most bone implants do not have antimicrobial capacity per se, pathogens are prone to construct biofilms on their surfaces, causing bacterial adherence and decreasing patient immunity and the efficacy of drug therapy. According to statistics, the probability of implant infection after orthopedic repair surgery is about 1–2%, and even reaches 5–10% in some high-risk patients, which affects patients’ postoperative recovery and increases the economic burden [137,138]. Therefore, antimicrobial modification of bone implant surfaces has become an important research topic. The surface is modified by electrochemical anodizing, plasma spraying techniques, topology modification, and bactericidal molecular coatings to affect the attachment and growth of bacteria. Zhao et al. [139] developed a PLGA-SDS/LA composite coating on the surface of ZA6-1 zinc alloy, achieving highly effective antibacterial activity through the synergistic action of SDS and LA. This coating demonstrated antibacterial efficacy of up to 98.9% against Staphylococcus aureus and 99.8% against Escherichia coli. The coating also maintained excellent osteoblast compatibility, offering a surface modification strategy for zinc-based orthopedic implants that combines preservative, antibacterial, and bioactive properties. Zhang et al. [140] developed a composite coating on titanium surfaces incorporating TiO_2_ nanorods/curcumin/HA/bone morphogenetic protein-2 onto a titanium surface. Using a 1060 nm NIR-II laser under mild conditions at 45 °C for 15 min, they achieved biofilm clearance, providing a low-temperature phototherapy solution for deep implant infections. Rao et al. [141] developed a Ti-MXene bone implant material triggered by near-infrared light, combined a Ti3C2TX MXene coating with a titanium substrate via electrostatic self-adsorption, and encapsulated it with gelatin. Under 808 nm near-infrared irradiation, photothermal antibacterial effects were realized, reactive oxygen species were effectively scavenged, bacterial biofilm formation was inhibited, and inflammatory responses were mitigated. In addition to engineered surface modification, the intrinsic degradation process of biodegradable materials forms a dynamic interface, which affects bacterial adhesion and biofilm formation. Using 3D imaging technology with a resolution below 40 nm, it was revealed that the Mg degradation layer has a complex nanoporous network and continuously changing morphology [61]. Figure 20 shows that the magnesium alloy has a variety of residual metals after being implanted in the body for nine months. The degradation process leads to the continuous release of Mg ions, affecting the local chemical environment [142]. This unstable morphology and chemical environment may affect bacterial attachment. He et al. [143] constructed a 3D porous Fe@Zn scaffold via electrodeposition technology. Leveraging the rapid release of zinc ions, it demonstrated ≥95% antibacterial efficacy against Staphylococcus aureus and nearly 100% efficacy against Escherichia coli in vitro.

The above surface functionalization modification strategies endow implants with efficient and proactive antimicrobial capabilities, which can reduce the chances of postoperative bone implant infections. These strategies can be briefly categorized into composite coatings combined with antibacterial ions, photothermal/sonodynamic synergistic therapy, etc. Meanwhile, surface coatings containing osteogenic factors are an important direction for next-generation bone implants to construct an integrated platform of “anti-infection and pro-repair”. For example, Zeng et al. [144] developed a Ti/PDA/BP composite coating by combining polydopamine and black phosphorus nanosheets. Under the synergistic action of near-infrared light and ultrasound, this coating eliminated 96.6% of Staphylococcus aureus in vitro and 97.3% in vivo within 10 min, endowing titanium implants with dual functions of infection prevention and enhanced tissue repair.

### 5.3. Osteointegration Effect

Osteointegration is the direct contact between the implant and the patient’s own bone tissue to form a functional connection. In addition to surface antimicrobial properties, osseointegration is a key performance indicator in the field of orthopedic therapy, where incomplete integration of the implant-bone interface is a major cause of surgical failure [145]. Bone implants commonly used in clinical practice are still deficient in promoting bone tissue regeneration and interfacial integration. Therefore, researchers have focused on surface modification by changing the physical topology and introducing bioactive coatings to provide excellent osteogenic properties and good biocompatibility. Bai et al. [146] transplanted the targeting peptide sequence and osteogenic growth peptide derived from bone marrow mesenchymal stem cells onto the surface of titanium implants. A biomimetic coating based on mussel-derived peptides was designed to induce directed osteogenesis of endogenous BMSCs under osteoporotic conditions, thereby enhancing osseointegration and improving biocompatibility. Liu et al. [147] developed a biomimetic layered scaffold coated with HAp on its surface. In vivo studies confirmed that it promoted angiogenesis and heterotopic bone formation within 4 weeks, and induced new bone matrix formation at sites distant from the interface by 8 weeks, providing a simple and efficient strategy for bone regeneration. Zhang et al. [148] developed a multifunctional TiO_2_@UCN/Qr/LA coating on a titanium surface. Through the synergistic action of quercetin (Qr) and nitric oxide (NO), bone tumors were ablated at 48 °C, Staphylococcus aureus biofilms were cleared at 45 °C, and angiogenesis and osteogenic differentiation were promoted.

**Figure 21:**
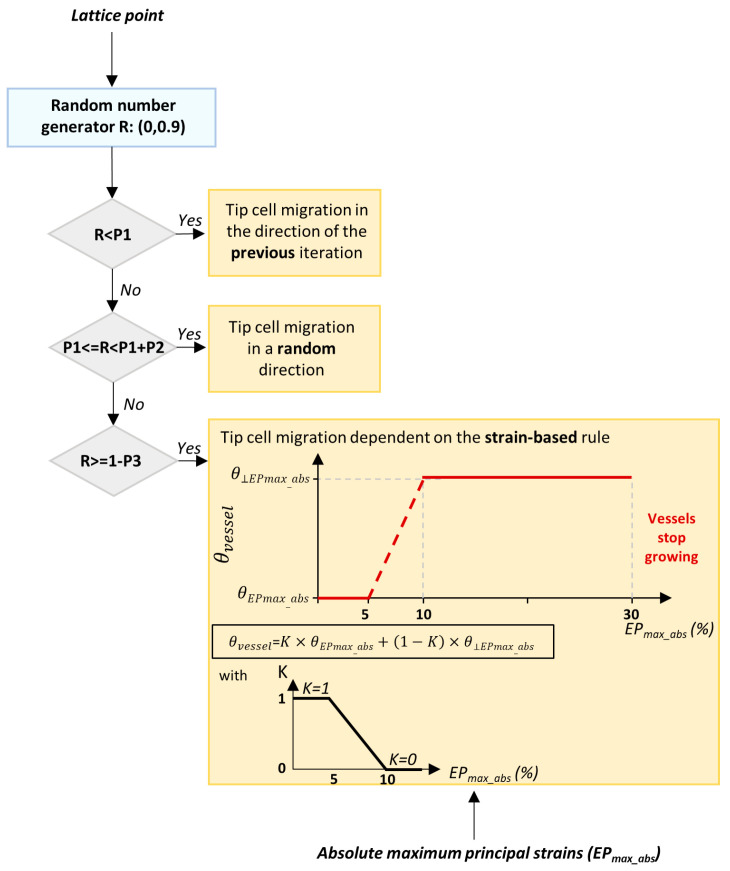
Algorithm implemented to simulate vessel growth. (Reproduced from [149] with permission).

Beyond biochemical cues, the mechanical environment established by the implant is a critical regulator of bone regeneration, particularly in steering the crucial process of angiogenesis. Recent research provides a compelling mechanistic link between the mechanical performance of an implant and its biological outcomes. Dazzi et al. [149] designed an algorithm for simulating vascular growth, as shown in Figure 21. It was demonstrated that external mechanical loads can “cover” the mechanical communication between cells and directly regulate the germination behavior of endothelial cells during early bone regeneration. Therefore, optimizing mechanical environments such as stiffness and porous structures can effectively transfer physiological loads and actively promote angiogenesis. Raffa et al. [150] established a two-dimensional finite element model of the bone-implant interface, systematically investigating the effects of surface roughness, material stiffness, and bone-implant contact ratio on the stress field within the peri-implant bone tissue. It was found that a low contact ratio exacerbates stress shielding, while increasing the contact ratio effectively reduces local stress shielding and promotes osseointegration.

Achieving rapid and robust osseointegration between the implant and the host bone is one of the measures of success in orthopedic surgery. By integrating the latest advancements in molecular biology, biomimetic materials science, and biomechanics, bone implant surfaces have undergone multifunctional and intelligent modifications. This has become the core driving force in solving osseointegration challenges and advancing the development of next-generation implants. In the future, active surfaces with integrated interfacial self-healing capabilities are projected to be a key direction for research.

## 6. Application of AI in the Field of Bone Implants

Current research in the field of bone implants primarily focuses on ideal bone graft structures, common material properties, immunomodulation techniques, and their subsequent application outcomes [151]. While the previous text thoroughly explored the key technologies for achieving personalization (medical imaging, additive manufacturing, etc.), these technologies are become relatively mature. Bone graft research is further advanced by cutting-edge science and intelligent computing, with artificial intelligence (AI) and machine learning (ML) serving as core tools.

**Figure 22:**
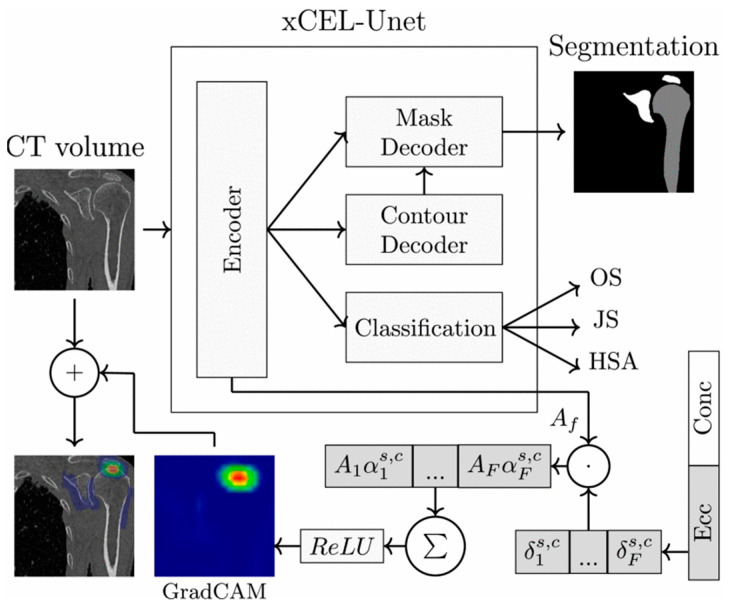
xCEL-UNet model. (Reproduced from [154] with permission).

Inspired by the AlphaFold project in the field of protein structure prediction, deep learning methods can also be applied to the orthopedic field. Generative AI and open science platforms such as The Open Molecules 2025 are powering the revolution in biomaterials. These tools can improve research efficiency and reduce trial and error costs. In fact, AI has already advanced multiple stages of the bone grafting process. AI models can be trained based on a patient’s biomarker features and defect characteristics to predict the optimal scaffold microstructure that can trigger specific cellular responses [152]. Convolutional Neural Networks (CNN), a deep learning model, can achieve AI-assisted segmentation and registration. By mixing the transformer network into CNN, semi-automatic segmentation of musculoskeletal images is achieved [153]. Marsilio et al. [154] innovatively introduced a deep learning network xCEL-Unet to achieve joint image segmentation and pathological evaluation, as shown in Figure 22. ML algorithms are being integrated to accelerate finite element (FE) simulations. These AI-driven models can predict stress distribution and bone remodeling results faster, thereby iteratively optimizing the mechanical environment of implants in the FE model of the patient’s bones [155]. Statistical shape modeling (SSM) is an important method for determining the average morphology and specificity of bones. For example, a large amount of relevant data and ML techniques have been used to quantify the morphological variations in the distal tibia, providing data support for subsequent replacement surgery [156].

**Figure 23:**
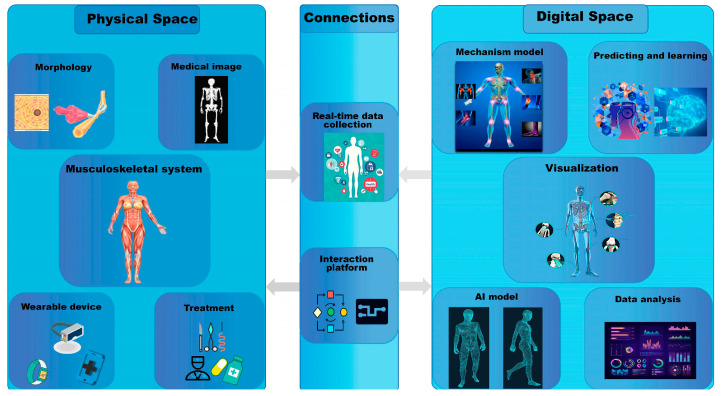
Digital twin of the whole musculoskeletal system for personalized diagnosis and treatment. (Reproduced from [157] with permission).

The integration of AI with real-world data has driven the application of digital twins in the medical field. virtual dynamic copies of patient physiological data and medical devices can be created, enabling advance planning, real-time monitoring, and postoperative simulation. Sun et al. [157] innovatively applied digital twins to the musculoskeletal system and established a 3D virtual reality system, as shown in Figure 23. It can reflect the real-time state of the human lumbar spine, enabling the prevention and monitoring of spinal diseases. Ahn et al. [158] developed a digital twin system based on a parametric reduced-order model, helping to evaluate and optimize the placement of dental implants. This transition from static implants to dynamic smart systems is a significant direction in the development of personalized medicine.

## 7. Conclusions and Perspectives

Research progress on personalized bone implants based on additive manufacturing has been systematically summarized, including design methodologies, material systems, fabrication techniques, and performance evaluation. Bone implants have made significant progress in creating anatomical matches and functionality. However, the issue of long-term in vivo performance and safety monitoring still exists in clinical applications. Especially for degradable materials such as magnesium and polymers, degradation products and wear debris pose potential hazards. The shift from standardized production to patient-specific customization of implants also poses challenges to the existing regulatory system. The integration of artificial intelligence, machine learning, and biomedical engineering provides opportunities and pathways to overcome these challenges.

(1) Machine learning models can be trained on long-term clinical follow-up data, which can be used to predict patient implant lifespan and identify risk factors. Furthermore, machine learning models can simulate the systematic distribution and biological effects of degradation products, transforming safety assessments from post-analysis to proactive design.
(2) A digital twin virtual platform can overcome regulatory bottlenecks for personalized implants. Implants equipped with biosensors can feed patient physiological data back to their digital twin. This feedback loop can achieve real-time detection of postoperative status, prevent the occurrence of complications, and customize personalized rehabilitation plans.

In the future, the development of the personalized bone implant field may not be limited to improvements in materials or printing technology but rather depend on the strategic integration of advanced manufacturing, data-driven, and intelligent technologies.

## Figures and Tables

**Table 1:** Comparative analysis of three design methods.

	Constructive Solid Geometry	Topology Optimization	Triply Periodic Minimal Surfaces
Design Principles	Constructing complex structures through Boolean operations on basic geometric primitives	Simulation-driven material distribution under specified constraints to achieve optimal performance	Generation of Periodic Porous Structures Based on Implicit Mathematical Surfaces
Core Advantages	Simple operation, rapid generation, and easy implementation of gradient structures	Structural lightweighting, optimal mechanical properties, low stress shielding effect	Structures exhibit continuous smoothness, excellent pore connectivity, and high biocompatibility
Limitations	Structural connectivity and mechanical properties are inferior to TPMS and topology optimization	Complex design process with high computational costs, requiring integration of manufacturing constraints	Mathematical modeling is relatively complex; gradient design requires integration with other methods
Primary Applications	Bionic gradient structures and personalized implants	Lightweight implant design, load-bearing structure optimization, multi-objective performance design	Promotes osseointegration, features highly permeable structures, and mimics biological systems
Development Trends	Combined with bioactive coatings, smart materials, and multi-material printing	Multiphysics optimization, biodegradable material design, AI-assisted	Gradient TPMS, topology optimization fusion, multiscale modeling

**Table 2:** Comparison of metallic, polymeric, and ceramic materials.

Material Type	Representative Materials	Advantages	Disadvantages	Scope of Application
Metallic Materials	Titanium and its alloys	Excellent biocompatibility, low elastic modulus, high strength, corrosion resistance	Poor wear resistance, relatively high cost	Load-bearing implants: joint prosthesis stems, bone plates, screws, spinal fusion devices
	Cobalt-chromium alloys	Exceptional wear resistance, highest strength and hardness, corrosion resistance	Excessively high elastic modulus, relatively poor biocompatibility, allergenic potential	Friction pairs in joint replacements: femoral heads and femoral condyles in artificial hip
	Stainless steel (316L)	Low cost, easy machinability, good mechanical properties	Worst corrosion resistance, high elastic modulus, relatively poorest biocompatibility	Primarily used for temporary implants: fracture fixation pins, plates, screws
	Biodegradable alloys	Biodegradable and absorbable, elastic modulus closest to bone, degradation products promote osteogenesis	Difficult to control degradation rate, potential hydrogen gas generation during degradation	Fracture internal fixation in non-weight-bearing areas: cardiovascular stents, maxillofacial
Ceramic Materials	Alumina/zirconia	Optimal biocompatibility, highest wear resistance, corrosion resistance	High brittleness, difficult to process and manufacture, high cost	Wear-resistant interfaces in joint replacements: Femoral heads and acetabular liners in hip joints
	Hydroxyapatite	Excellent osteoconductivity, capable of forming chemical bonds with bone	High brittleness, low strength, unsuitable for load-bearing applications	Surface coatings for metal implants, bone defect fillers
	β-tricalcium phosphate	High bioactivity, promotes bone products serve as osteogenic raw materials	High brittleness, low strength, degradation rate requires control	Bone defect filling: Spinal fusion, bone cysts, alveolar ridge augmentation
Polymeric Materials	Ultra-high molecular weight polyethylene	Exceptional toughness, good wear resistance, low friction coefficient	Creep behavior, wear particles may induce bone resorption	Wear-bearing surfaces in joint replacements: Acetabular liners, tibial pads
	Polyether ether ketone	Low elastic modulus, radiolucent, fatigue-resistant, high machinability	Lacks osseointegration capability, average wear resistance, low strength	Spinal fusion devices, non-load-bearing components for joint replacements

**Table 3:** Comparison of five 3D printing technologies for bone implant material applications.

	SLM	EBM	FDM	BJ	SLA
Working Principle	Laser selective complete melting of metal powder layers	Electron beam melts preheated metal powder layers in vacuum	Heated nozzles melt and extrude polymer filaments, layering them sequentially	The nozzle sprays binder onto the powder bed, followed by sintering to densify the part	Ultraviolet light selectively cures the flowing slurry and undergoes post-processing
Core Advantages	High precision, high density, mechanical properties close to forgings	Low residual stress, fast printing speed, clean vacuum environment	Extremely low cost, simple operation, diverse materials	Extremely fast printing speed, capable of producing highly complex structures, no thermal stress	High resolution, good surface finish, high density
Primary Disadvantages	Significant residual stresses, high equipment and material costs, surface requires polishing	Rougher surface, relatively lower precision, extremely high equipment and maintenance costs	Poor precision and surface quality, risk of microbial growth	Complex post-processing, high shrinkage rate, mechanical properties depend on sintering quality	Complex post-processing, Limitations of materials and slurries
Typical Materials	Ti6Al4V, CoCr, 316L stainless steel	Ti6Al4V, pure titanium	PEEK, PLA	Titanium powder, calcium phosphate ceramics	Ceramics, polymers
Surface Quality	Rough surface (requires post-processing)	Very rough (requires post-processing)	Noticeable layer lines, rough surface	Depends on powder and sintering process	Good surface finish
Primary Applications	Load-bearing permanent implants (hip, knee)	Load-bearing permanent implants (large low-stress components)	Surgical guides, planning models, surface coatings	Complex porous bone scaffolds, non-load-bearing implants	Dental, craniofacial, and orthopedic

## Data Availability

The original contributions presented in this study are included in the article. Further inquiries can be directed to the corresponding author(s).

## Abbreviations

The following abbreviations are used in this manuscript:

CT	computed tomography
Mimics	Materialise’s interactive medical image control system
L-PBF	Laser Powder Bed Fusion
SLM	Selective Laser Melting
AI	artificial intelligence
CSG	Constructive Solid Geometry
TPMS	Triple Periodic Minimal Surface
LSRCMS	layered rod-connected hexagonal porous structure
NPR	Negative Poisson’s Ratio
OC	optimality criteria
HA/HAp	hydroxyapatite
PLA	polylactic acid
β-TCP	β-tricalcium phosphate
BCP	biphasic calcium phosphate
Mg	magnesium
Fe	iron
Zn	zinc
PEEK	polyether ether ketone
UHMWPE	ultra-high molecular weight polyethylene
PDA	poly-dopamine
Mn	manganese
Cu	copper
nMgO	nano-magnesium oxide
PLLA	left-handed polylactic acid
ATZ	alumina toughened zirconia
CPC	calcium phosphate ceramics
BAG	bioactive glass
TTCP	tricalcium phosphate
WH	Whitlockite
PMMA	polymethyl methacrylate
PCL	polycaprolactone
EBM	Electron Beam Melting
FDM	Fused Deposition Modeling
BJ	Binder Jetting
PBF	Powder Bed Fusion
SLS	Selective Laser Sintering
MJF	Multi-Jet Fusion
SLA	Stereolithography
DO	diamond-like
RD	rhombic dodecahedral
PLGA	polylactic-polyhydroxyacetic acid copolymer
PA	polyamide
Qr	quercetin
NO	nitric oxide
ML	machine learning
CNN	Convolutional Neural Networks
FE	finite element
SSM	statistical shape modeling
